# Supplementary material for: SUMOylation Protects FASN Against Proteasomal Degradation in Breast Cancer Cells Treated with Grape Leaf Extract
Source: Biomolecules. 2020 Mar 31;10(4):529. doi: 10.3390/biom10040529 (PMC7226518; doi:10.3390/biom10040529)
Supplement: Supplementary file 1 [file biomolecules-10-00529-s001.zip › Supplementary files/Supplementary Figure 2.pptx]

## Slide 1
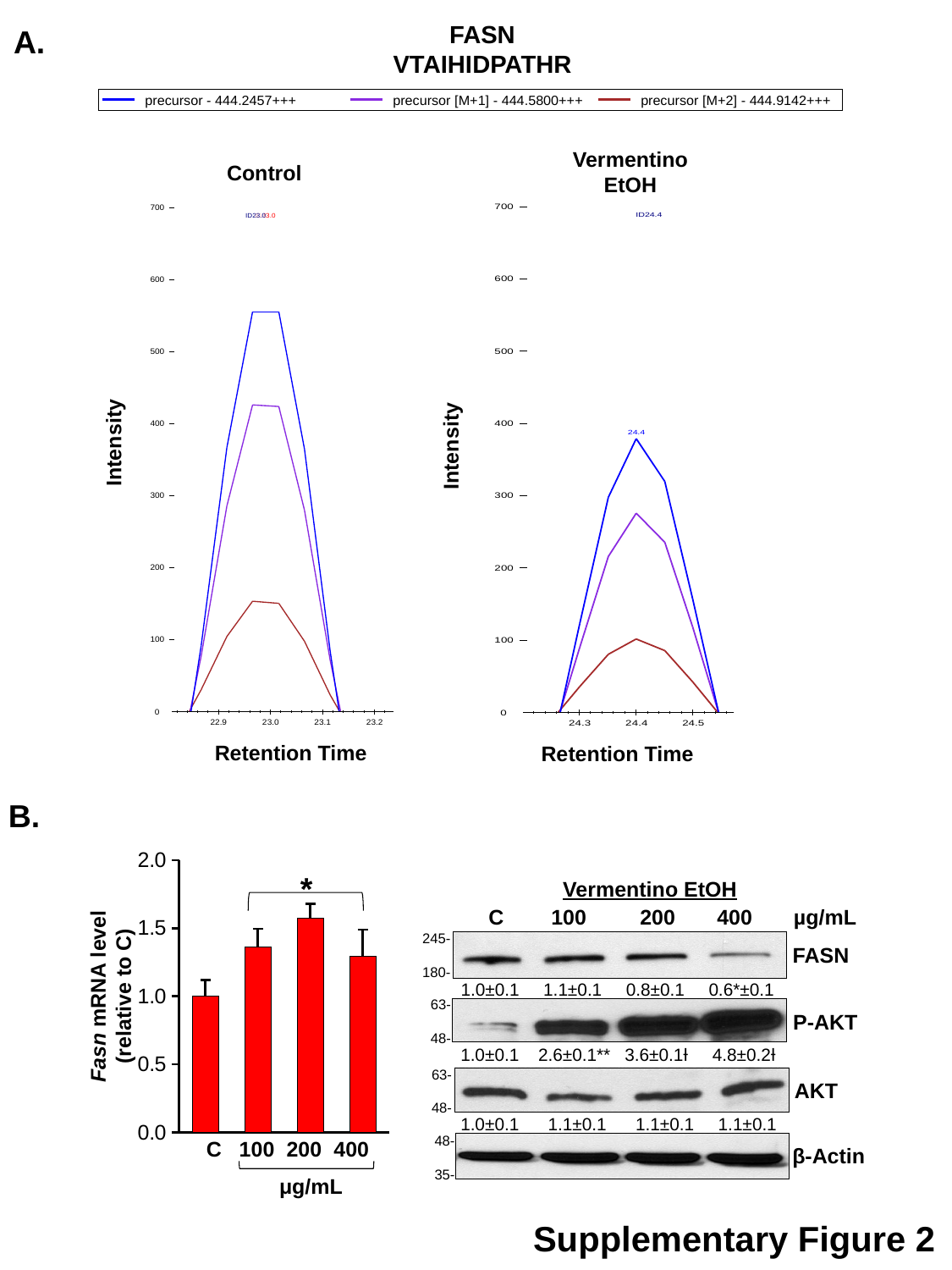

FASN
VTAIHIDPATHR
A.
Vermentino
EtOH
Control
Intensity
Intensity
Retention Time
Retention Time
B.
### Chart
| Category | FASN |
|---|---|
| 0 | 1.0 |
| 100 | 1.3634916202096374 |
| 200 | 1.5755866477584308 |
| 400 | 1.2960832822772028 |*
Fasn mRNA level
(relative to C)
C 100 200 400
μg/mL
Vermentino EtOH
C 100 200 400 µg/mL
245-
180-
FASN
1.0±0.1 1.1±0.1 0.8±0.1 0.6*±0.1
63-
48-
P-AKT
1.0±0.1 2.6±0.1** 3.6±0.1Ɨ 4.8±0.2Ɨ
63-
48-
AKT
1.0±0.1 1.1±0.1 1.1±0.1 1.1±0.1
48-
35-
β-Actin
Supplementary Figure 2
