# Supplementary material for: SUMOylation Protects FASN Against Proteasomal Degradation in Breast Cancer Cells Treated with Grape Leaf Extract
Source: Biomolecules. 2020 Mar 31;10(4):529. doi: 10.3390/biom10040529 (PMC7226518; doi:10.3390/biom10040529)
Supplement: Supplementary file 1 [file biomolecules-10-00529-s001.zip › Supplementary files/Supplementary Figure 3.pptx]

## Slide 1
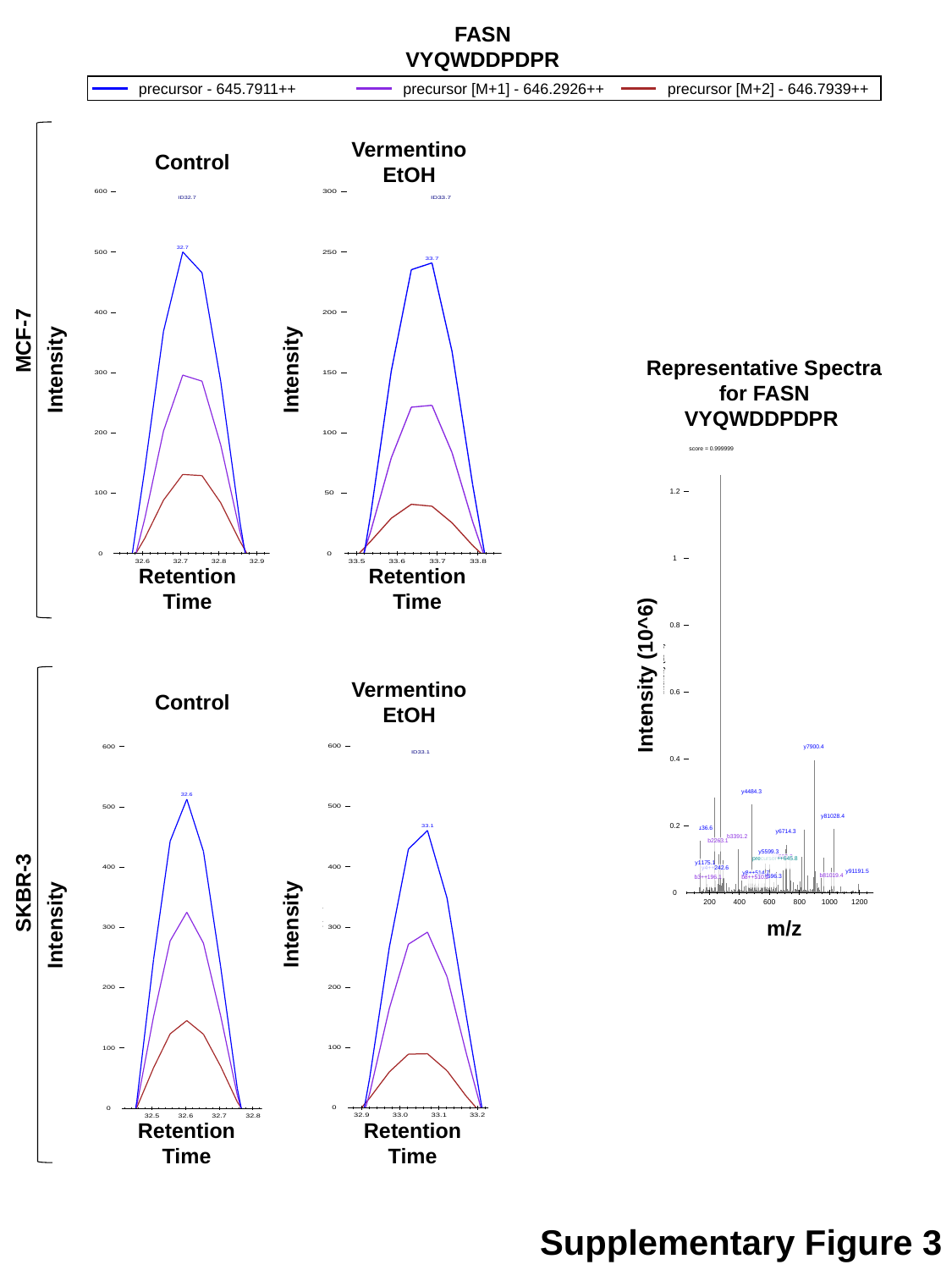

FASN
VYQWDDPDPR
Vermentino
EtOH
Control
Intensity
Intensity
MCF-7
Representative Spectra for FASN
VYQWDDPDPR
Retention Time
Retention Time
Intensity (10^6)
Vermentino
EtOH
Control
Intensity
Intensity
SKBR-3
m/z
Retention Time
Retention Time
Supplementary Figure 3
