# Supplementary material for: SUMOylation Protects FASN Against Proteasomal Degradation in Breast Cancer Cells Treated with Grape Leaf Extract
Source: Biomolecules. 2020 Mar 31;10(4):529. doi: 10.3390/biom10040529 (PMC7226518; doi:10.3390/biom10040529)
Supplement: Supplementary file 1 [file biomolecules-10-00529-s001.zip › Supplementary files/Supplementary Figure 4.pptx]

## Slide 1
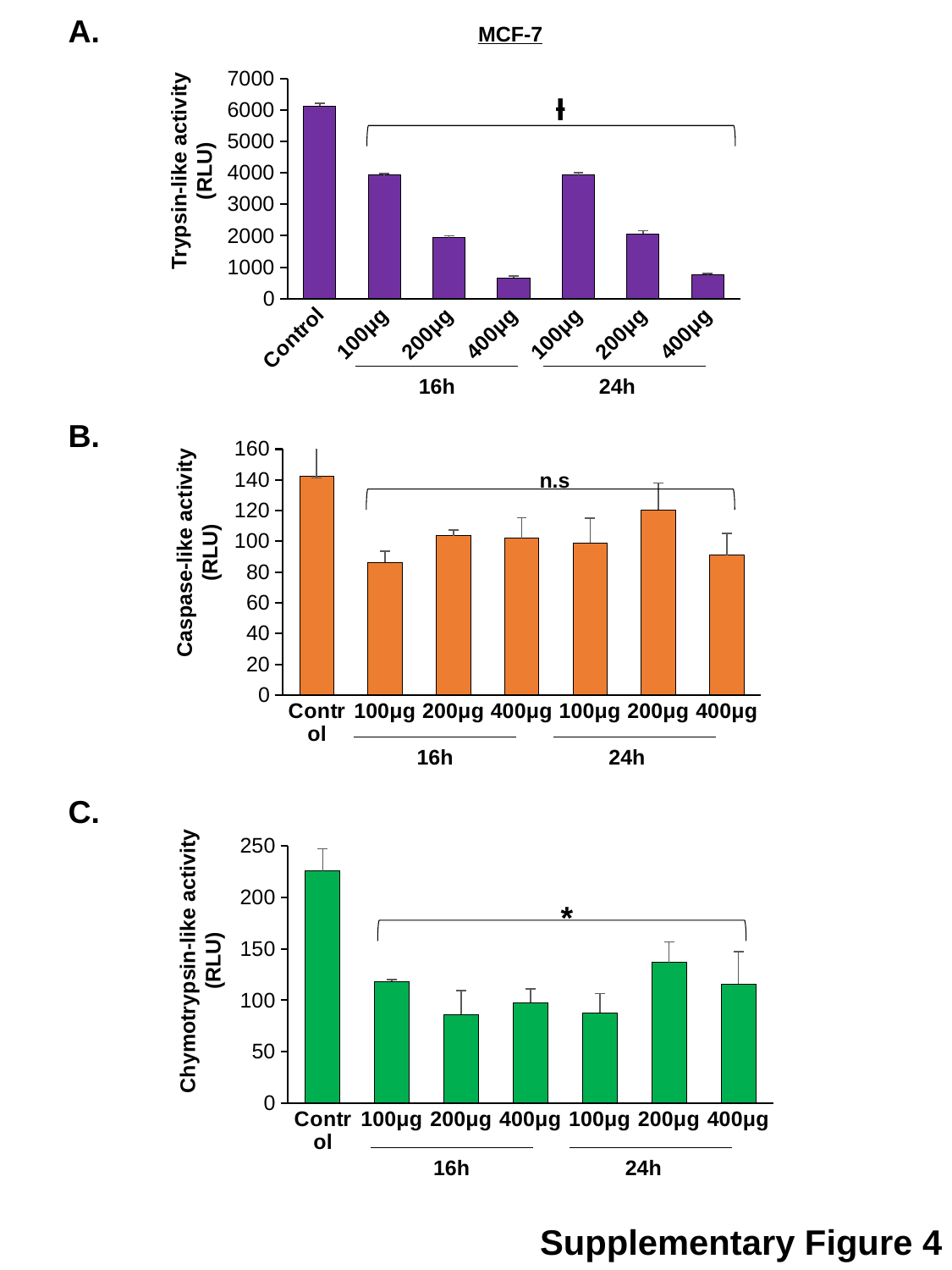

A.
MCF-7
### Chart
| Category | |
|---|---|
| Control | 6115.833333333333 |
| 100μg | 3929.0 |
| 200μg | 1933.0 |
| 400μg | 661.6666666666666 |
| 100μg | 3933.0 |
| 200μg | 2054.3333333333335 |
| 400μg | 761.0 |Ɨ
Trypsin-like activity
(RLU)
16h
24h
B.
### Chart
| Category | |
|---|---|
| Control | 142.16666666666666 |
| 100μg | 86.33333333333333 |
| 200μg | 103.66666666666667 |
| 400μg | 102.0 |
| 100μg | 99.0 |
| 200μg | 120.33333333333333 |
| 400μg | 91.0 |n.s
Caspase-like activity
(RLU)
16h
24h
C.
### Chart
| Category | |
|---|---|
| Control | 225.33333333333334 |
| 100μg | 118.33333333333333 |
| 200μg | 86.0 |
| 400μg | 97.66666666666667 |
| 100μg | 87.66666666666667 |
| 200μg | 136.66666666666666 |
| 400μg | 115.66666666666667 |*
Chymotrypsin-like activity
(RLU)
16h
24h
Supplementary Figure 4
