# Supplementary material for: SUMOylation Protects FASN Against Proteasomal Degradation in Breast Cancer Cells Treated with Grape Leaf Extract
Source: Biomolecules. 2020 Mar 31;10(4):529. doi: 10.3390/biom10040529 (PMC7226518; doi:10.3390/biom10040529)
Supplement: Supplementary file 1 [file biomolecules-10-00529-s001.zip › Supplementary files/Supplementary Figure 5.pptx]

## Slide 1
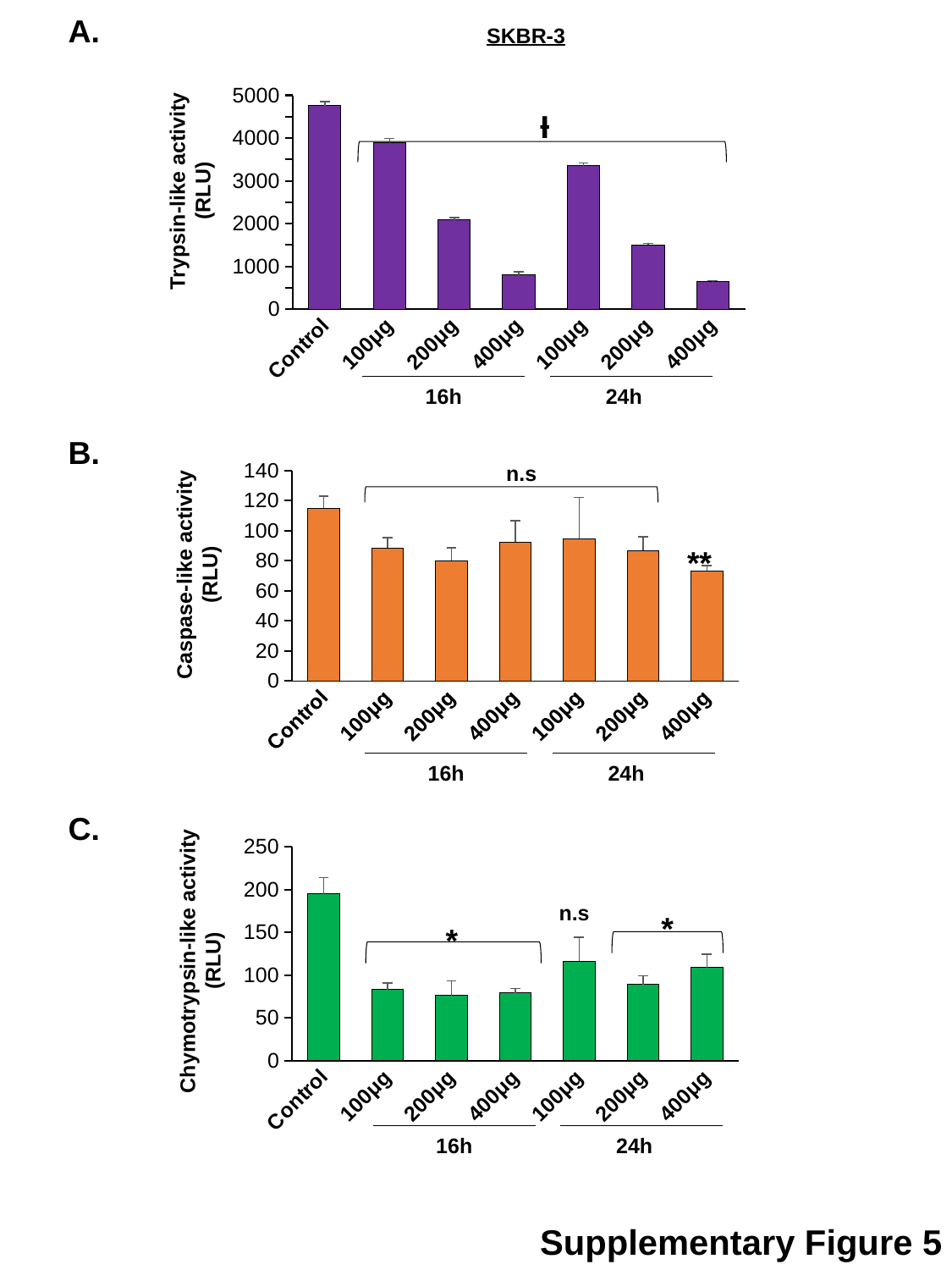

A.
SKBR-3
### Chart
| Category | |
|---|---|
| Control | 4754.333333333333 |
| 100μg | 3889.3333333333335 |
| 200μg | 2082.3333333333335 |
| 400μg | 802.6666666666666 |
| 100μg | 3356.0 |
| 200μg | 1504.0 |
| 400μg | 639.6666666666666 |Ɨ
Trypsin-like activity
(RLU)
16h
24h
B.
n.s
### Chart
| Category | |
|---|---|
| Control | 115.0 |
| 100μg | 88.33333333333333 |
| 200μg | 80.0 |
| 400μg | 92.33333333333333 |
| 100μg | 94.66666666666667 |
| 200μg | 86.66666666666667 |
| 400μg | 73.33333333333333 |**
Caspase-like activity
(RLU)
16h
24h
C.
### Chart
| Category | |
|---|---|
| Control | 194.83333333333334 |
| 100μg | 83.33333333333333 |
| 200μg | 76.0 |
| 400μg | 79.0 |
| 100μg | 116.33333333333333 |
| 200μg | 89.0 |
| 400μg | 108.66666666666667 |
n.s
*
*
Chymotrypsin-like activity
(RLU)
16h
24h
Supplementary Figure 5
