# Supplementary material for: SUMOylation Protects FASN Against Proteasomal Degradation in Breast Cancer Cells Treated with Grape Leaf Extract
Source: Biomolecules. 2020 Mar 31;10(4):529. doi: 10.3390/biom10040529 (PMC7226518; doi:10.3390/biom10040529)
Supplement: Supplementary file 1 [file biomolecules-10-00529-s001.zip › Supplementary files/SUPPLEMENTARY FIGURE LEGENDS.docx]

**SUPPLEMENTARY FIGURE LEGENDS**

**Supplementary Figure 1. Vermentino hydroalcoholic extract alters the proteomic profile in MCF-7 and SKBR-3 cell lines** Canonical pathways from ingenuity pathway analysis (IPA) gene ontology algorithms of dysregulated proteins associated with Vermentino hydroalcoholic extract treatment (24 hours) in MCF-7 and SKBR-3 cell lines.

**Supplementary Figure 2. Vermentino hydroalcoholic extract lowers FASN protein level in SKBR-3 cells.** Cells were treated with 100ug/mL, 200ug/mL, and 400ug/mL Vermentino hydroalcoholic extract for 24 hours. **(A)** Intensity of the extracted precursor isotopic envelope (M, M+1, M+2) of a representative FASN peptide VTAIHIDPATHR. All match the theoretical isotopic distribution. **(B)** mRNA level of FASN was accomplished by using RT-PCR, while protein level was analyzed by Western blot analysis for FASN, AKT and p-AKT. Densitometric ratios normalized to β-actin are shown below the western blot. Results are expressed as a fold of control (mean±SE) from 4 and 3 independent experiments performed in triplicate for mRNA and proteins analysis, respectively. *P<0.05, **P<0.002, ^Ɨ^P<0.001 vs control.

**Supplementary Figure 3.** **Vermentino hydroalcoholic and hydro extracts lower FASN protein level in MCF-7 and SKBR-3 cell lines. (Top and bottom left panels)** Intensity of the extracted precursor isotopic envelope (M, M+1, M+2) of a representative FASN peptide VYQWDDPDPR in SKBR3 and MCF7 cells treated with Vermentino hydroalcoholic (EtOH) extract. All match the theoretical isotopic distribution. **(right panel)** Representative spectra of the unique FASN peptide, VYQWDDPDPR.

**Supplementary Figure 4. Vermentino hydroalcoholic and hydro extracts inhibit trypsin-like and chymotrypsin-like proteasomal degradation in MCF-7 cells.** Cells were treated with 100ug/mL, 200ug/mL, and 400ug/mL Vermentino hydroalcoholic (EtOH) and hydro (H2O) extracts for 24 hours. (A) Trypsin-like, (B) Caspase-like, (C) Chymotrypsin-like of 20S proteasome activities were detected as the relative light unit (RLU) generated from the cleaved substrate in the reagent. (mean ± SEM) from 3 independent experiments. *P<0.05; **P<0.005; †P <0.0001 vs. control.

**Supplementary Figure 5. Vermentino hydroalcoholic and hydro extracts inhibit trypsin-like and chymotrypsin-like proteasomal degradation in SKBR-3 cells.** Cells were treated with 100ug/mL, 200ug/mL, and 400ug/mL Vermentino hydroalcoholic (EtOH) and hydro (H2O) extracts for 24 hours. (A) Trypsin-like, (B) Caspase-like, (C) Chymotrypsin-like of 20S proteasome activities were detected as the relative light unit (RLU) generated from the cleaved substrate in the reagent. (mean ± SEM) from 3 independent experiments. *P<0.05; **P<0.005; †P < 0.0001 vs. control.
