# Supplementary material for: SUMOylation Protects FASN Against Proteasomal Degradation in Breast Cancer Cells Treated with Grape Leaf Extract
Source: Biomolecules. 2020 Mar 31;10(4):529. doi: 10.3390/biom10040529 (PMC7226518; doi:10.3390/biom10040529)
Supplement: Supplementary file 1 [file biomolecules-10-00529-s001.zip › Supplementary files/supplementary Table 1.docx]

|  |  |  |  |  |  |  | **Pathologic (PN)** | | **ER** | | **PR** | | **ErbB2** | |  |  | |  | |
| --- | --- | --- | --- | --- | --- | --- | --- | --- | --- | --- | --- | --- | --- | --- | --- | --- | --- | --- | --- |
| **n** | **age** | **T** | **size** | **grade** | **M0** | **N0** | **grade** | **LN** | +/- | **grade** | +/- | **grade** | +/- | **grade** | **CK** | | **Ki67** | |  |
| Tumor breast tissue 1 | 47 | DCIS | pT1 (1.2cm) | 2 | M0 | N0 | pN0 |  | + | 90% | + | 90% | + | 3 | - | | 30% | |  |
| Tumor breast tissue 2 | 66 | DCIS | pT1 (1.5cm) | 2 | M0 | N0 | pN+ | N/A | + | 90% | + | 90% | + | 2 | - | | 10% | |  |
| Tumor breast tissue 3 | 64 | DCIS | pT1 (1.7cm) | 2 | M0 | N0 | pN0 |  | + | 85% | + | 80% | + | 2 | - | | 5% | |  |
| Tumor breast tissue 4 | 70 | DCIS | pT2 (3cm ) | 3 | M0 | N0 | pN+ | LN 2/4 | + | 90% | + | 80% | + | 2 | + | | 20% | |  |
| Tumor breast tissue 5 | 52 | DCIS | pT1 (1.5cm) | 2 | M0 | N0 | pN0 |  | + | 90% | + | 20% | + | 2 | - | | 10% | |  |
| Tumor breast tissue 6 | 68 | DCIS | pT1 (1.5cm) | 2 | M0 | N0 | pN+ | LN 1/6 | + | 90% | + | 65% | + | 2 | + | | 5% | |  |
| Tumor breast tissue 7 | 84 | IDC | pT1 (1cm) | 2 | M0 | N0 | pN0 |  | + | 90% | + | 1% | + | 2 | - | | 18% | |  |
| Tumor breast tissue 8 | 56 | IDC | pT2 (2.8cm) | 3 | M0 | N0 | pN+ | LN 7/14 | + | 90% | + | 20% | + | 3 | + | | 40% | |  |
| Tumor breast tissue 9 | 66 | DCIS | pT1 (0.8cm) | 2 | M0 | N0 | pN0 |  | + | 90% | + | 80% | + | 3 | - | | 5% | |  |
| Tumor breast tissue 10 | 43 | IDC | pT1 (2cm) | 2 | M0 | N0 | pN+mic | LN 1 mic /8 | + | 90% | + | 80% | + | 3 | + | | 5% | |  |
| Normal breast tissue 1 | 41 |  |  |  |  |  |  |  |  |  |  |  |  |  |  | |  | |  |
| Normal breast tissue 2 | 48 |  |  |  |  |  |  |  |  |  |  |  |  |  |  | |  | |  |
| Normal breast tissue 3 | 32 |  |  |  |  |  |  |  |  |  |  |  |  |  |  | |  | |  |
| Normal breast tissue 4 | 39 |  |  |  |  |  |  |  |  |  |  |  |  |  |  | |  | |  |
| Normal breast tissue 5 | 52 |  |  |  |  |  |  |  |  |  |  |  |  |  |  | |  | |  |

**Supplementary Table 1. Characteristics of breast cancer tissues from ErbB2-positive patients.**
