# Supplementary material for: SUMOylation Protects FASN Against Proteasomal Degradation in Breast Cancer Cells Treated with Grape Leaf Extract
Source: Biomolecules. 2020 Mar 31;10(4):529. doi: 10.3390/biom10040529 (PMC7226518; doi:10.3390/biom10040529)
Supplement: Supplementary file 1 [file biomolecules-10-00529-s001.zip › Supplementary files/Supplementary Table 2.docx]

| **Supplementary table 2. LIST OF PROTEINS OF VERMENTINO ETOH EXTRACT-TREATED MCF-7 CELLS** | | |  |
| --- | --- | --- | --- |
|  |  |  |  |
| **Symbol** | **Entrez Gene Name** | **GenPept/UniProt/Swiss-Prot Accession** | **Expr Log Ratio** |
| **PTPN12** | protein tyrosine phosphatase, non-receptor type 12 | Q05209 | -5 |
| **SERBP1** | SERPINE1 mRNA binding protein 1 | Q8NC51 | -5 |
| **DOP1B** | DOP1 leucine zipper like protein B | Q9Y3R5 | -5 |
| **ATAD3A** | ATPase family AAA domain containing 3A | Q9NVI7 | -5 |
| **PPP4R3A** | protein phosphatase 4 regulatory subunit 3A | Q6IN85 | -5 |
| **KLC1** | kinesin light chain 1 | Q07866 | -5 |
| **SLC25A12** | solute carrier family 25 member 12 | O75746 | -5 |
| **NUP205** | nucleoporin 205 | Q92621 | -5 |
| **VAMP8** | vesicle associated membrane protein 8 | Q9BV40 | -5 |
| **XRCC1** | X-ray repair cross complementing 1 | P18887 | -5 |
| **METAP2** | methionyl aminopeptidase 2 | P50579 | -5 |
| **MYO18A** | myosin XVIIIA | Q92614 | -5 |
| **LUC7L2** | LUC7 like 2, pre-mRNA splicing factor | Q9Y383 | -5 |
| **SMARCA5** | SWI/SNF related, matrix associated, actin dependent regulator of chromatin, subfamily a, member 5 | O60264 | -5 |
| **KYAT3** | kynurenine aminotransferase 3 | Q6YP21 | -5 |
| **STK10** | serine/threonine kinase 10 | O94804 | -5 |
| **NCAPD2** | non-SMC condensin I complex subunit D2 | Q15021 | -5 |
| **TCEA1** | transcription elongation factor A1 | P23193 | -5 |
| **GSTK1** | glutathione S-transferase kappa 1 | Q9Y2Q3 | -5 |
| **SEPT11** | septin 11 | Q9NVA2 | -5 |
| **PPP1R7** | protein phosphatase 1 regulatory subunit 7 | Q15435 | -5 |
| **S100A4** | S100 calcium binding protein A4 | P26447 | -5 |
| **TCERG1** | transcription elongation regulator 1 | O14776 | -5 |
| **CARMIL1** | capping protein regulator and myosin 1 linker 1 | Q5VZK9 | -5 |
| **TXNRD2** | thioredoxin reductase 2 | Q9NNW7 | -5 |
| **SLIRP** | SRA stem-loop interacting RNA binding protein | Q9GZT3 | -5 |
| **GSK3A** | glycogen synthase kinase 3 alpha | P49840 | -5 |
| **BPTF** | bromodomain PHD finger transcription factor | Q12830 | -5 |
| **RPS6KA1** | ribosomal protein S6 kinase A1 | Q15418 | -5 |
| **U2SURP** | U2 snRNP associated SURP domain containing | O15042 | -5 |
| **THOC2** | THO complex 2 | Q8NI27 | -5 |
| **EIF3C** | eukaryotic translation initiation factor 3 subunit C | Q99613 | -5 |
| **SNRPF** | small nuclear ribonucleoprotein polypeptide F | P62306 | -5 |
| **CGN** | cingulin | Q9P2M7 | -5 |
| **CLU** | clusterin | P10909 | -5 |
| **HGH1** | HGH1 homolog | Q9BTY7 | -5 |
| **GATAD2B** | GATA zinc finger domain containing 2B | Q8WXI9 | -5 |
| **SFPQ** | splicing factor proline and glutamine rich | P23246 | -3.684 |
| **FAM98B** | family with sequence similarity 98 member B | Q52LJ0 | -3.297 |
| **DCPS** | decapping enzyme, scavenger | Q96C86 | -3.273 |
| **EDC4** | enhancer of mRNA decapping 4 | Q6P2E9 | -3.163 |
| **ACSL3** | acyl-CoA synthetase long chain family member 3 | O95573 | -3.03 |
| **CPSF7** | cleavage and polyadenylation specific factor 7 | Q8N684 | -2.921 |
| **EWSR1** | EWS RNA binding protein 1 | Q01844 | -2.921 |
| **EPS8L1** | EPS8 like 1 | Q8TE68 | -2.892 |
| **UBE2I** | ubiquitin conjugating enzyme E2 I | P63279 | -2.891 |
| **CSTF3** | cleavage stimulation factor subunit 3 | Q12996 | -2.818 |
| **CBS/CBSL** | cystathionine-beta-synthase | P0DN79 | -2.805 |
| **FUS** | FUS RNA binding protein | P35637 | -2.753 |
| **MOCS3** | molybdenum cofactor synthesis 3 | O95396 | -2.749 |
| **SGPL1** | sphingosine-1-phosphate lyase 1 | O95470 | -2.723 |
| **COPG2** | coatomer protein complex subunit gamma 2 | Q9UBF2 | -2.652 |
| **NONO** | non-POU domain containing octamer binding | Q15233 | -2.569 |
| **SF3A3** | splicing factor 3a subunit 3 | Q12874 | -2.527 |
| **YBX1** | Y-box binding protein 1 | P67809 | -2.524 |
| **RBM3** | RNA binding motif protein 3 | P98179 | -2.508 |
| **CPSF6** | cleavage and polyadenylation specific factor 6 | Q16630 | -2.419 |
| **POLR2B** | RNA polymerase II subunit B | P30876 | -2.419 |
| **SCYL1** | SCY1 like pseudokinase 1 | Q96KG9 | -2.323 |
| **FLAD1** | flavin adenine dinucleotide synthetase 1 | Q8NFF5 | -2.293 |
| **MYBBP1A** | MYB binding protein 1a | Q9BQG0 | -2.231 |
| **GLRX3** | glutaredoxin 3 | O76003 | -2.225 |
| **PABPC4** | poly(A) binding protein cytoplasmic 4 | Q13310 | -2.203 |
| **ZC3H4** | zinc finger CCCH-type containing 4 | Q9UPT8 | -2.177 |
| **KRT10** | keratin 10 | P13645 | -2.121 |
| **RPL22L1** | ribosomal protein L22 like 1 | Q6P5R6 | -2.09 |
| **HS1BP3** | HCLS1 binding protein 3 | Q53T59 | -2.019 |
| **UBE2O** | ubiquitin conjugating enzyme E2 O | Q9C0C9 | -2.004 |
| **RAB8A** | RAB8A, member RAS oncogene family | P61006 | -1.997 |
| **G3BP1** | G3BP stress granule assembly factor 1 | Q13283 | -1.952 |
| **EPCAM** | epithelial cell adhesion molecule | P16422 | -1.926 |
| **KHDRBS1** | KH RNA binding domain containing, signal transduction associated 1 | Q07666 | -1.912 |
| **NDRG3** | NDRG family member 3 | Q9UGV2 | -1.905 |
| **SYNCRIP** | synaptotagmin binding cytoplasmic RNA interacting protein | O60506 | -1.903 |
| **KTN1** | kinectin 1 | Q86UP2 | -1.894 |
| **SF3A1** | splicing factor 3a subunit 1 | Q15459 | -1.821 |
| **DDX56** | DEAD-box helicase 56 | Q9NY93 | -1.796 |
| **ALB** | albumin | P02768 | -1.757 |
| **TRIP6** | thyroid hormone receptor interactor 6 | Q15654 | -1.751 |
| **DDX5** | DEAD-box helicase 5 | P17844 | -1.733 |
| **FKBP2** | FKBP prolyl isomerase 2 | P26885 | -1.707 |
| **HNRNPUL2** | heterogeneous nuclear ribonucleoprotein U like 2 | Q1KMD3 | -1.699 |
| **SHMT1** | serine hydroxymethyltransferase 1 | P34896 | -1.698 |
| **L2HGDH** | L-2-hydroxyglutarate dehydrogenase | Q9H9P8 | -1.696 |
| **HK2** | hexokinase 2 | P52789 | -1.691 |
| **STXBP2** | syntaxin binding protein 2 | Q15833 | -1.69 |
| **NXF1** | nuclear RNA export factor 1 | Q9UBU9 | -1.689 |
| **DDAH1** | dimethylarginine dimethylaminohydrolase 1 | O94760 | -1.68 |
| **EFTUD2** | elongation factor Tu GTP binding domain containing 2 | Q15029 | -1.669 |
| **ERMP1** | endoplasmic reticulum metallopeptidase 1 | Q7Z2K6 | -1.66 |
| **PPP6C** | protein phosphatase 6 catalytic subunit | O00743 | -1.648 |
| **CYFIP1** | cytoplasmic FMR1 interacting protein 1 | Q7L576 | -1.646 |
| **CNBP** | CCHC-type zinc finger nucleic acid binding protein | P62633 | -1.633 |
| **GRHPR** | glyoxylate and hydroxypyruvate reductase | Q9UBQ7 | -1.628 |
| **RBM47** | RNA binding motif protein 47 | A0AV96 | -1.611 |
| **HNRNPUL1** | heterogeneous nuclear ribonucleoprotein U like 1 | Q9BUJ2 | -1.594 |
| **LSM2** | LSM2 homolog, U6 small nuclear RNA and mRNA degradation associated | Q9Y333 | -1.585 |
| **TGM2** | transglutaminase 2 | P21980 | -1.551 |
| **RTCB** | RNA 2',3'-cyclic phosphate and 5'-OH ligase | Q9Y3I0 | -1.535 |
| **PLCB3** | phospholipase C beta 3 | Q01970 | -1.533 |
| **TRIP12** | thyroid hormone receptor interactor 12 | Q14669 | -1.533 |
| **DNMT1** | DNA methyltransferase 1 | P26358 | -1.526 |
| **HNRNPDL** | heterogeneous nuclear ribonucleoprotein D like | O14979 | -1.521 |
| **MAPK1** | mitogen-activated protein kinase 1 | P28482 | -1.499 |
| **DSTN** | destrin, actin depolymerizing factor | P60981 | -1.494 |
| **GRWD1** | glutamate rich WD repeat containing 1 | Q9BQ67 | -1.492 |
| **FKBP15** | FKBP prolyl isomerase 15 | Q5T1M5 | -1.464 |
| **PSPC1** | paraspeckle component 1 | Q8WXF1 | -1.455 |
| **SPTBN2** | spectrin beta, non-erythrocytic 2 | O15020 | -1.423 |
| **PPP2R5E** | protein phosphatase 2 regulatory subunit B'epsilon | Q16537 | -1.397 |
| **TNKS1BP1** | tankyrase 1 binding protein 1 | Q9C0C2 | -1.39 |
| **EIF6** | eukaryotic translation initiation factor 6 | P56537 | -1.388 |
| **CHMP1A** | charged multivesicular body protein 1A | Q9HD42 | -1.383 |
| **ABCE1** | ATP binding cassette subfamily E member 1 | P61221 | -1.377 |
| **S100A14** | S100 calcium binding protein A14 | Q9HCY8 | -1.356 |
| **HNRNPK** | heterogeneous nuclear ribonucleoprotein K | P61978 | -1.327 |
| **NUP107** | nucleoporin 107 | P57740 | -1.325 |
| **SET** | SET nuclear proto-oncogene | Q01105 | -1.302 |
| **BCLAF1** | BCL2 associated transcription factor 1 | Q9NYF8 | -1.301 |
| **DDX3X** | DEAD-box helicase 3 X-linked | O00571 | -1.299 |
| **RPL22** | ribosomal protein L22 | P35268 | -1.278 |
| **AP1S1** | adaptor related protein complex 1 subunit sigma 1 | P61966 | -1.262 |
| **HNRNPU** | heterogeneous nuclear ribonucleoprotein U | Q00839 | -1.26 |
| **PRPF4** | pre-mRNA processing factor 4 | O43172 | -1.24 |
| **SRRT** | serrate, RNA effector molecule | Q9BXP5 | -1.239 |
| **NDC1** | NDC1 transmembrane nucleoporin | Q9BTX1 | -1.233 |
| **SULT2B1** | sulfotransferase family 2B member 1 | O00204 | -1.226 |
| **KRT1** | keratin 1 | P04264 | -1.215 |
| **ERO1A** | endoplasmic reticulum oxidoreductase 1 alpha | Q96HE7 | -1.204 |
| **EIF5A** | eukaryotic translation initiation factor 5A | P63241 | -1.2 |
| **RANBP2** | RAN binding protein 2 | P49792 | -1.182 |
| **KRT2** | keratin 2 | P35908 | -1.178 |
| **SRP68** | signal recognition particle 68 | Q9UHB9 | -1.169 |
| **RBM39** | RNA binding motif protein 39 | Q14498 | -1.157 |
| **DDX17** | DEAD-box helicase 17 | Q92841 | -1.149 |
| **RSL1D1** | ribosomal L1 domain containing 1 | O76021 | -1.146 |
| **EIF2B3** | eukaryotic translation initiation factor 2B subunit gamma | Q9NR50 | -1.134 |
| **POP1** | POP1 homolog, ribonuclease P/MRP subunit | Q99575 | -1.125 |
| **PREP** | prolyl endopeptidase | P48147 | -1.11 |
| **XRN2** | 5'-3' exoribonuclease 2 | Q9H0D6 | -1.106 |
| **RPS27A** | ribosomal protein S27a | P62979 | -1.105 |
| **DARS** | aspartyl-tRNA synthetase | P14868 | -1.093 |
| **DNAJA2** | DnaJ heat shock protein family (Hsp40) member A2 | O60884 | -1.078 |
| **RRM1** | ribonucleotide reductase catalytic subunit M1 | P23921 | -1.076 |
| **SRPRA** | SRP receptor subunit alpha | P08240 | -1.066 |
| **DDX46** | DEAD-box helicase 46 | Q7L014 | -1.061 |
| **NME2** | NME/NM23 nucleoside diphosphate kinase 2 | P22392 | -1.056 |
| **LPCAT1** | lysophosphatidylcholine acyltransferase 1 | Q8NF37 | -1.054 |
| **SNRPD2** | small nuclear ribonucleoprotein D2 polypeptide | P62316 | -1.052 |
| **SPAG9** | sperm associated antigen 9 | O60271 | -1.047 |
| **RTRAF** | RNA transcription, translation and transport factor | Q9Y224 | -1.047 |
| **CALR** | calreticulin | P27797 | -1.043 |
| **HSP90AB2P** | heat shock protein 90 alpha family class B member 2, pseudogene | Q58FF8 | -1.035 |
| **MAP2K1** | mitogen-activated protein kinase kinase 1 | Q02750 | -1.014 |
| **NAT10** | N-acetyltransferase 10 | Q9H0A0 | -1.01 |
| **CNP** | 2',3'-cyclic nucleotide 3' phosphodiesterase | P09543 | -1.008 |
| **BSG** | basigin (Ok blood group) | P35613 | -1.007 |
| **HSPA4** | heat shock protein family A (Hsp70) member 4 | P34932 | -1.006 |
| **DENR** | density regulated re-initiation and release factor | O43583 | -1.006 |
| **SMC2** | structural maintenance of chromosomes 2 | O95347 | -1.005 |
| **ME1** | malic enzyme 1 | P48163 | -1 |
| **HECTD3** | HECT domain E3 ubiquitin protein ligase 3 | Q5T447 | -0.986 |
| **OGDH** | oxoglutarate dehydrogenase | Q02218 | -0.971 |
| **ACSF3** | acyl-CoA synthetase family member 3 | Q4G176 | -0.969 |
| **EPHX1** | epoxide hydrolase 1 | P07099 | -0.964 |
| **AIP** | aryl hydrocarbon receptor interacting protein | O00170 | -0.962 |
| **SRPK2** | SRSF protein kinase 2 | P78362 | -0.936 |
| **RPL10** | ribosomal protein L10 | P27635 | -0.935 |
| **TJP2** | tight junction protein 2 | Q9UDY2 | -0.933 |
| **PREX1** | phosphatidylinositol-3,4,5-trisphosphate dependent Rac exchange factor 1 | Q8TCU6 | -0.926 |
| **MCM3** | minichromosome maintenance complex component 3 | P25205 | -0.916 |
| **PTGES2** | prostaglandin E synthase 2 | Q9H7Z7 | -0.908 |
| **MYO6** | myosin VI | Q9UM54 | -0.898 |
| **PKP3** | plakophilin 3 | Q9Y446 | -0.894 |
| **POLR1C** | RNA polymerase I and III subunit C | O15160 | -0.886 |
| **TBCE** | tubulin folding cofactor E | Q15813 | -0.882 |
| **TMX2** | thioredoxin related transmembrane protein 2 | Q9Y320 | -0.881 |
| **LTA4H** | leukotriene A4 hydrolase | P09960 | -0.879 |
| **PAPSS2** | 3'-phosphoadenosine 5'-phosphosulfate synthase 2 | O95340 | -0.856 |
| **RDH13** | retinol dehydrogenase 13 | Q8NBN7 | -0.855 |
| **S100A8** | S100 calcium binding protein A8 | P05109 | -0.852 |
| **PLOD3** | procollagen-lysine,2-oxoglutarate 5-dioxygenase 3 | O60568 | -0.849 |
| **DCTN1** | dynactin subunit 1 | Q14203 | -0.847 |
| **DNAAF5** | dynein axonemal assembly factor 5 | Q86Y56 | -0.845 |
| **RBBP7** | RB binding protein 7, chromatin remodeling factor | Q16576 | -0.839 |
| **PSMD3** | proteasome 26S subunit, non-ATPase 3 | O43242 | -0.831 |
| **CLIC1** | chloride intracellular channel 1 | O00299 | -0.83 |
| **RPL38** | ribosomal protein L38 | P63173 | -0.828 |
| **THRAP3** | thyroid hormone receptor associated protein 3 | Q9Y2W1 | -0.825 |
| **PPAT** | phosphoribosyl pyrophosphate amidotransferase | Q06203 | -0.823 |
| **CORO1A** | coronin 1A | P31146 | -0.819 |
| **CHMP3** | charged multivesicular body protein 3 | Q9Y3E7 | -0.818 |
| **PARP1** | poly(ADP-ribose) polymerase 1 | P09874 | -0.816 |
| **HSP90AB1** | heat shock protein 90 alpha family class B member 1 | P08238 | -0.813 |
| **PGRMC1** | progesterone receptor membrane component 1 | O00264 | -0.807 |
| **API5** | apoptosis inhibitor 5 | Q9BZZ5 | -0.805 |
| **ARL3** | ADP ribosylation factor like GTPase 3 | P36405 | -0.804 |
| **RPS11** | ribosomal protein S11 | P62280 | -0.804 |
| **EDF1** | endothelial differentiation related factor 1 | O60869 | -0.799 |
| **IGBP1** | immunoglobulin binding protein 1 | P78318 | -0.795 |
| **LMNB1** | lamin B1 | P20700 | -0.795 |
| **PLEC** | plectin | Q15149 | -0.79 |
| **CRABP2** | cellular retinoic acid binding protein 2 | P29373 | -0.79 |
| **NSUN2** | NOP2/Sun RNA methyltransferase family member 2 | Q08J23 | -0.787 |
| **NUMA1** | nuclear mitotic apparatus protein 1 | Q14980 | -0.786 |
| **ALYREF** | Aly/REF export factor | Q86V81 | -0.785 |
| **WASL** | Wiskott-Aldrich syndrome like | O00401 | -0.784 |
| **SNRNP70** | small nuclear ribonucleoprotein U1 subunit 70 | P08621 | -0.781 |
| **SORD** | sorbitol dehydrogenase | Q00796 | -0.773 |
| **IST1** | IST1 factor associated with ESCRT-III | P53990 | -0.773 |
| **SLC1A5** | solute carrier family 1 member 5 | Q15758 | -0.768 |
| **BCAS2** | BCAS2 pre-mRNA processing factor | O75934 | -0.767 |
| **TXNRD1** | thioredoxin reductase 1 | Q16881 | -0.763 |
| **DNM1L** | dynamin 1 like | O00429 | -0.761 |
| **RPL30** | ribosomal protein L30 | P62888 | -0.757 |
| **ACTN4** | actinin alpha 4 | O43707 | -0.757 |
| **SPTAN1** | spectrin alpha, non-erythrocytic 1 | Q13813 | -0.747 |
| **DAZAP1** | DAZ associated protein 1 | Q96EP5 | -0.746 |
| **FAF2** | Fas associated factor family member 2 | Q96CS3 | -0.743 |
| **HEATR6** | HEAT repeat containing 6 | Q6AI08 | -0.74 |
| **ELAC2** | elaC ribonuclease Z 2 | Q9BQ52 | -0.735 |
| **RPL19** | ribosomal protein L19 | P84098 | -0.734 |
| **EIF2AK2** | eukaryotic translation initiation factor 2 alpha kinase 2 | P19525 | -0.728 |
| **TEX10** | testis expressed 10 | Q9NXF1 | -0.721 |
| **SRSF3** | serine and arginine rich splicing factor 3 | P84103 | -0.715 |
| **SUPT16H** | SPT16 homolog, facilitates chromatin remodeling subunit | Q9Y5B9 | -0.714 |
| **NCL** | nucleolin | P19338 | -0.713 |
| **CPNE3** | copine 3 | O75131 | -0.711 |
| **RDX** | radixin | P35241 | -0.707 |
| **AHSG** | alpha 2-HS glycoprotein | P02765 | -0.705 |
| **BCAS1** | breast carcinoma amplified sequence 1 | O75363 | -0.703 |
| **DNM2** | dynamin 2 | P50570 | -0.698 |
| **UBR4** | ubiquitin protein ligase E3 component n-recognin 4 | Q5T4S7 | -0.696 |
| **USP14** | ubiquitin specific peptidase 14 | P54578 | -0.693 |
| **USP10** | ubiquitin specific peptidase 10 | Q14694 | -0.687 |
| **PAICS** | phosphoribosylaminoimidazole carboxylase and phosphoribosylaminoimidazolesuccinocarboxamide synthase | P22234 | -0.687 |
| **HGS** | hepatocyte growth factor-regulated tyrosine kinase substrate | O14964 | -0.683 |
| **IPO5** | importin 5 | O00410 | -0.679 |
| **TBL2** | transducin beta like 2 | Q9Y4P3 | -0.672 |
| **MCM4** | minichromosome maintenance complex component 4 | P33991 | -0.671 |
| **EIF3D** | eukaryotic translation initiation factor 3 subunit D | O15371 | -0.668 |
| **DYNC1LI2** | dynein cytoplasmic 1 light intermediate chain 2 | O43237 | -0.666 |
| **SEC13** | SEC13 homolog, nuclear pore and COPII coat complex component | P55735 | -0.664 |
| **LMNB2** | lamin B2 | Q03252 | -0.662 |
| **SARS2** | seryl-tRNA synthetase 2, mitochondrial | Q9NP81 | -0.651 |
| **FIS1** | fission, mitochondrial 1 | Q9Y3D6 | -0.65 |
| **RCC2** | regulator of chromosome condensation 2 | Q9P258 | -0.646 |
| **VARS** | valyl-tRNA synthetase | P26640 | -0.644 |
| **CORO1C** | coronin 1C | Q9ULV4 | -0.643 |
| **ESRP1** | epithelial splicing regulatory protein 1 | Q6NXG1 | -0.639 |
| **CKAP4** | cytoskeleton associated protein 4 | Q07065 | -0.638 |
| **GLG1** | golgi glycoprotein 1 | Q92896 | -0.637 |
| **U2AF2** | U2 small nuclear RNA auxiliary factor 2 | P26368 | -0.632 |
| **DHRS2** | dehydrogenase/reductase 2 | Q13268 | -0.63 |
| **POLD1** | DNA polymerase delta 1, catalytic subunit | P28340 | -0.629 |
| **SEC63** | SEC63 homolog, protein translocation regulator | Q9UGP8 | -0.629 |
| **RPA1** | replication protein A1 | P27694 | -0.627 |
| **RANGAP1** | Ran GTPase activating protein 1 | P46060 | -0.625 |
| **IARS** | isoleucyl-tRNA synthetase | P41252 | -0.618 |
| **SF3B1** | splicing factor 3b subunit 1 | O75533 | -0.617 |
| **NSFL1C** | NSFL1 cofactor | Q9UNZ2 | -0.614 |
| **PEBP1** | phosphatidylethanolamine binding protein 1 | P30086 | -0.613 |
| **RPL28** | ribosomal protein L28 | P46779 | -0.611 |
| **RPS23** | ribosomal protein S23 | P62266 | -0.605 |
| **HNRNPR** | heterogeneous nuclear ribonucleoprotein R | O43390 | -0.605 |
| **EDC3** | enhancer of mRNA decapping 3 | Q96F86 | -0.603 |
| **DHX15** | DEAH-box helicase 15 | O43143 | -0.602 |
| **RCC1** | regulator of chromosome condensation 1 | P18754 | -0.594 |
| **ALDH5A1** | aldehyde dehydrogenase 5 family member A1 | P51649 | -0.584 |
| **CDK1** | cyclin dependent kinase 1 | P06493 | -0.581 |
| **CCAR2** | cell cycle and apoptosis regulator 2 | Q8N163 | -0.581 |
| **MBOAT7** | membrane bound O-acyltransferase domain containing 7 | Q96N66 | -0.574 |
| **XRCC5** | X-ray repair cross complementing 5 | P13010 | -0.574 |
| **NDUFA7** | NADH:ubiquinone oxidoreductase subunit A7 | O95182 | -0.572 |
| **TRIM28** | tripartite motif containing 28 | Q13263 | -0.571 |
| **DHTKD1** | dehydrogenase E1 and transketolase domain containing 1 | Q96HY7 | -0.564 |
| **NCKAP1** | NCK associated protein 1 | Q9Y2A7 | -0.561 |
| **FBP2** | fructose-bisphosphatase 2 | O00757 | -0.555 |
| **GREB1** | growth regulating estrogen receptor binding 1 | Q4ZG55 | -0.554 |
| **NDUFB9** | NADH:ubiquinone oxidoreductase subunit B9 | Q9Y6M9 | -0.553 |
| **PITRM1** | pitrilysin metallopeptidase 1 | Q5JRX3 | -0.551 |
| **SH3GLB2** | SH3 domain containing GRB2 like, endophilin B2 | Q9NR46 | -0.55 |
| **RPL24** | ribosomal protein L24 | P83731 | -0.55 |
| **ECPAS** | Ecm29 proteasome adaptor and scaffold | Q5VYK3 | -0.546 |
| **SF1** | splicing factor 1 | Q15637 | -0.545 |
| **LMNA** | lamin A/C | P02545 | -0.54 |
| **CAPNS1** | calpain small subunit 1 | P04632 | -0.538 |
| **ARMT1** | acidic residue methyltransferase 1 | Q9H993 | -0.538 |
| **GART** | phosphoribosylglycinamide formyltransferase, phosphoribosylglycinamide synthetase, phosphoribosylaminoimidazole synthetase | P22102 | -0.538 |
| **NUP153** | nucleoporin 153 | P49790 | -0.537 |
| **TNPO1** | transportin 1 | Q92973 | -0.536 |
| **MBD3** | methyl-CpG binding domain protein 3 | O95983 | -0.535 |
| **PCYOX1** | prenylcysteine oxidase 1 | Q9UHG3 | -0.53 |
| **NUP93** | nucleoporin 93 | Q8N1F7 | -0.53 |
| **LMAN1** | lectin, mannose binding 1 | P49257 | -0.526 |
| **GDI1** | GDP dissociation inhibitor 1 | P31150 | -0.525 |
| **DBT** | dihydrolipoamide branched chain transacylase E2 | P11182 | -0.524 |
| **SNX2** | sorting nexin 2 | O60749 | -0.523 |
| **DIAPH1** | diaphanous related formin 1 | O60610 | -0.519 |
| **MESD** | mesoderm development LRP chaperone | Q14696 | -0.519 |
| **PSMD2** | proteasome 26S subunit, non-ATPase 2 | Q13200 | -0.519 |
| **PPIL1** | peptidylprolyl isomerase like 1 | Q9Y3C6 | -0.518 |
| **HNRNPH1** | heterogeneous nuclear ribonucleoprotein H1 | P31943 | -0.516 |
| **SF3B2** | splicing factor 3b subunit 2 | Q13435 | -0.514 |
| **SMARCE1** | SWI/SNF related, matrix associated, actin dependent regulator of chromatin, subfamily e, member 1 | Q969G3 | -0.513 |
| **USP5** | ubiquitin specific peptidase 5 | P45974 | -0.508 |
| **CPT1A** | carnitine palmitoyltransferase 1A | P50416 | -0.505 |
| **SND1** | staphylococcal nuclease and tudor domain containing 1 | Q7KZF4 | -0.504 |
| **PRKDC** | protein kinase, DNA-activated, catalytic subunit | P78527 | -0.5 |
| **BAG6** | BCL2 associated athanogene 6 | P46379 | -0.498 |
| **ANXA2** | annexin A2 | P07355 | -0.498 |
| **NCBP1** | nuclear cap binding protein subunit 1 | Q09161 | -0.497 |
| **HNRNPL** | heterogeneous nuclear ribonucleoprotein L | P14866 | -0.496 |
| **CAND1** | cullin associated and neddylation dissociated 1 | Q86VP6 | -0.493 |
| **CELF1** | CUGBP Elav-like family member 1 | Q92879 | -0.492 |
| **IPO4** | importin 4 | Q8TEX9 | -0.49 |
| **RPS20** | ribosomal protein S20 | P60866 | -0.49 |
| **GSS** | glutathione synthetase | P48637 | -0.489 |
| **LGALS3** | galectin 3 | P17931 | -0.484 |
| **ARPC1B** | actin related protein 2/3 complex subunit 1B | O15143 | -0.481 |
| **ARPC2** | actin related protein 2/3 complex subunit 2 | O15144 | -0.479 |
| **HNRNPA2B1** | heterogeneous nuclear ribonucleoprotein A2/B1 | P22626 | -0.478 |
| **XPO1** | exportin 1 | O14980 | -0.478 |
| **MCM6** | minichromosome maintenance complex component 6 | Q14566 | -0.476 |
| **SSRP1** | structure specific recognition protein 1 | Q08945 | -0.474 |
| **SNX1** | sorting nexin 1 | Q13596 | -0.474 |
| **ARF4** | ADP ribosylation factor 4 | P18085 | -0.469 |
| **NAA15** | N(alpha)-acetyltransferase 15, NatA auxiliary subunit | Q9BXJ9 | -0.469 |
| **TCP1** | t-complex 1 | P17987 | -0.469 |
| **TMEM205** | transmembrane protein 205 | Q6UW68 | -0.468 |
| **GAA** | glucosidase alpha, acid | P10253 | -0.464 |
| **GTF2I** | general transcription factor IIi | P78347 | -0.464 |
| **ACOX3** | acyl-CoA oxidase 3, pristanoyl | O15254 | -0.462 |
| **PPP2R1A** | protein phosphatase 2 scaffold subunit Aalpha | P30153 | -0.458 |
| **TARS** | threonyl-tRNA synthetase | P26639 | -0.454 |
| **PYCR3** | pyrroline-5-carboxylate reductase 3 | Q53H96 | -0.453 |
| **TUBB8** | tubulin beta 8 class VIII | Q3ZCM7 | -0.452 |
| **HK1** | hexokinase 1 | P19367 | -0.451 |
| **CSE1L** | chromosome segregation 1 like | P55060 | -0.451 |
| **DDX39B** | DExD-box helicase 39B | Q13838 | -0.45 |
| **GARS** | glycyl-tRNA synthetase | P41250 | -0.448 |
| **ANXA6** | annexin A6 | P08133 | -0.448 |
| **RPS19** | ribosomal protein S19 | P39019 | -0.445 |
| **PYCR1** | pyrroline-5-carboxylate reductase 1 | P32322 | -0.442 |
| **COPS8** | COP9 signalosome subunit 8 | Q99627 | -0.442 |
| **SLC2A1** | solute carrier family 2 member 1 | P11166 | -0.442 |
| **EIF5B** | eukaryotic translation initiation factor 5B | O60841 | -0.44 |
| **RO60** | Ro60, Y RNA binding protein | P10155 | -0.44 |
| **FKBP10** | FKBP prolyl isomerase 10 | Q96AY3 | -0.439 |
| **EIF4A3** | eukaryotic translation initiation factor 4A3 | P38919 | -0.438 |
| **CD9** | CD9 molecule | P21926 | -0.438 |
| **CDC37** | cell division cycle 37 | Q16543 | -0.434 |
| **ATP1B1** | ATPase Na+/K+ transporting subunit beta 1 | P05026 | -0.431 |
| **DDX1** | DEAD-box helicase 1 | Q92499 | -0.427 |
| **CSDE1** | cold shock domain containing E1 | O75534 | -0.426 |
| **SF3B3** | splicing factor 3b subunit 3 | Q15393 | -0.426 |
| **PDCD6** | programmed cell death 6 | O75340 | -0.424 |
| **GDPD3** | glycerophosphodiester phosphodiesterase domain containing 3 | Q7L5L3 | -0.423 |
| **CAPN1** | calpain 1 | P07384 | -0.423 |
| **PYCARD** | PYD and CARD domain containing | Q9ULZ3 | -0.423 |
| **HNRNPD** | heterogeneous nuclear ribonucleoprotein D | Q14103 | -0.42 |
| **RPS12** | ribosomal protein S12 | P25398 | -0.42 |
| **PNPT1** | polyribonucleotide nucleotidyltransferase 1 | Q8TCS8 | -0.419 |
| **SOD1** | superoxide dismutase 1 | P00441 | -0.419 |
| **EIF4G2** | eukaryotic translation initiation factor 4 gamma 2 | P78344 | -0.417 |
| **IARS2** | isoleucyl-tRNA synthetase 2, mitochondrial | Q9NSE4 | -0.416 |
| **RPL23** | ribosomal protein L23 | P62829 | -0.414 |
| **RPS18** | ribosomal protein S18 | P62269 | -0.414 |
| **MCTS1** | MCTS1 re-initiation and release factor | Q9ULC4 | -0.414 |
| **EIF4H** | eukaryotic translation initiation factor 4H | Q15056 | -0.413 |
| **ATL2** | atlastin GTPase 2 | Q8NHH9 | -0.412 |
| **CA2** | carbonic anhydrase 2 | P00918 | -0.41 |
| **PHF5A** | PHD finger protein 5A | Q7RTV0 | -0.409 |
| **DYNC1I2** | dynein cytoplasmic 1 intermediate chain 2 | Q13409 | -0.407 |
| **RAVER1** | ribonucleoprotein, PTB binding 1 | Q8IY67 | -0.407 |
| **F11R** | F11 receptor | Q9Y624 | -0.405 |
| **PGM2** | phosphoglucomutase 2 | Q96G03 | -0.403 |
| **ATP6V1C1** | ATPase H+ transporting V1 subunit C1 | P21283 | -0.403 |
| **SUCLG1** | succinate-CoA ligase alpha subunit | P53597 | -0.403 |
| **MTHFD1L** | methylenetetrahydrofolate dehydrogenase (NADP+ dependent) 1 like | Q6UB35 | -0.402 |
| **POLE3** | DNA polymerase epsilon 3, accessory subunit | Q9NRF9 | -0.402 |
| **IAH1** | isoamyl acetate hydrolyzing esterase 1 (putative) | Q2TAA2 | -0.401 |
| **LARP4B** | La ribonucleoprotein domain family member 4B | Q92615 | -0.399 |
| **CANX** | calnexin | P27824 | -0.399 |
| **IPO7** | importin 7 | O95373 | -0.398 |
| **PSMD5** | proteasome 26S subunit, non-ATPase 5 | Q16401 | -0.398 |
| **ARMCX3** | armadillo repeat containing X-linked 3 | Q9UH62 | -0.395 |
| **UBA2** | ubiquitin like modifier activating enzyme 2 | Q9UBT2 | -0.395 |
| **UBE2D3** | ubiquitin conjugating enzyme E2 D3 | P61077 | -0.395 |
| **SEPT7** | septin 7 | Q16181 | -0.392 |
| **ITGB1** | integrin subunit beta 1 | P05556 | -0.392 |
| **CTNNBL1** | catenin beta like 1 | Q8WYA6 | -0.389 |
| **SYAP1** | synapse associated protein 1 | Q96A49 | -0.388 |
| **PGRMC2** | progesterone receptor membrane component 2 | O15173 | -0.388 |
| **NOP9** | NOP9 nucleolar protein | Q86U38 | -0.387 |
| **DRG1** | developmentally regulated GTP binding protein 1 | Q9Y295 | -0.385 |
| **PDIA4** | protein disulfide isomerase family A member 4 | P13667 | -0.383 |
| **KRT9** | keratin 9 | P35527 | -0.382 |
| **MAP4** | microtubule associated protein 4 | P27816 | -0.381 |
| **RNH1** | ribonuclease/angiogenin inhibitor 1 | P13489 | -0.377 |
| **SNX12** | sorting nexin 12 | Q9UMY4 | -0.376 |
| **ALDH2** | aldehyde dehydrogenase 2 family member | P05091 | -0.376 |
| **NARS** | asparaginyl-tRNA synthetase | O43776 | -0.375 |
| **HSPH1** | heat shock protein family H (Hsp110) member 1 | Q92598 | -0.375 |
| **RPL9** | ribosomal protein L9 | P32969 | -0.373 |
| **PRKAR2A** | protein kinase cAMP-dependent type II regulatory subunit alpha | P13861 | -0.371 |
| **IQGAP1** | IQ motif containing GTPase activating protein 1 | P46940 | -0.37 |
| **TRAP1** | TNF receptor associated protein 1 | Q12931 | -0.37 |
| **AIFM1** | apoptosis inducing factor mitochondria associated 1 | O95831 | -0.369 |
| **DSP** | desmoplakin | P15924 | -0.369 |
| **KPNA2** | karyopherin subunit alpha 2 | P52292 | -0.365 |
| **EZR** | ezrin | P15311 | -0.363 |
| **ACTL6A** | actin like 6A | O96019 | -0.362 |
| **GNL1** | G protein nucleolar 1 (putative) | P36915 | -0.359 |
| **SRM** | spermidine synthase | P19623 | -0.355 |
| **CHD4** | chromodomain helicase DNA binding protein 4 | Q14839 | -0.354 |
| **RPRD1B** | regulation of nuclear pre-mRNA domain containing 1B | Q9NQG5 | -0.352 |
| **NDUFA5** | NADH:ubiquinone oxidoreductase subunit A5 | Q16718 | -0.349 |
| **AFDN** | afadin, adherens junction formation factor | P55196 | -0.348 |
| **ATIC** | 5-aminoimidazole-4-carboxamide ribonucleotide formyltransferase/IMP cyclohydrolase | P31939 | -0.348 |
| **SARS** | seryl-tRNA synthetase | P49591 | -0.347 |
| **FKBP4** | FKBP prolyl isomerase 4 | Q02790 | -0.346 |
| **CBX3** | chromobox 3 | Q13185 | -0.344 |
| **DHX9** | DExH-box helicase 9 | Q08211 | -0.342 |
| **TSFM** | Ts translation elongation factor, mitochondrial | P43897 | -0.34 |
| **PYGB** | glycogen phosphorylase B | P11216 | -0.337 |
| **SQOR** | sulfide quinone oxidoreductase | Q9Y6N5 | -0.334 |
| **PELP1** | proline, glutamate and leucine rich protein 1 | Q8IZL8 | -0.332 |
| **RPL13A** | ribosomal protein L13a | P40429 | -0.33 |
| **UBA1** | ubiquitin like modifier activating enzyme 1 | P22314 | -0.328 |
| **MRPS18B** | mitochondrial ribosomal protein S18B | Q9Y676 | -0.327 |
| **DDX39A** | DExD-box helicase 39A | O00148 | -0.327 |
| **NCLN** | nicalin | Q969V3 | -0.324 |
| **TFAM** | transcription factor A, mitochondrial | Q00059 | -0.322 |
| **NUBP2** | nucleotide binding protein 2 | Q9Y5Y2 | -0.318 |
| **RPS13** | ribosomal protein S13 | P62277 | -0.318 |
| **PMVK** | phosphomevalonate kinase | Q15126 | -0.316 |
| **XRCC6** | X-ray repair cross complementing 6 | P12956 | -0.316 |
| **RAB5B** | RAB5B, member RAS oncogene family | P61020 | -0.315 |
| **MYH14** | myosin heavy chain 14 | Q7Z406 | -0.313 |
| **THUMPD3** | THUMP domain containing 3 | Q9BV44 | -0.313 |
| **EIF5** | eukaryotic translation initiation factor 5 | P55010 | -0.313 |
| **ATP2A2** | ATPase sarcoplasmic/endoplasmic reticulum Ca2+ transporting 2 | P16615 | -0.312 |
| **SEPT9** | septin 9 | Q9UHD8 | -0.311 |
| **XPO5** | exportin 5 | Q9HAV4 | -0.311 |
| **BAIAP2** | BAI1 associated protein 2 | Q9UQB8 | -0.31 |
| **SDHA** | succinate dehydrogenase complex flavoprotein subunit A | P31040 | -0.31 |
| **MYOF** | myoferlin | Q9NZM1 | -0.31 |
| **TBRG4** | transforming growth factor beta regulator 4 | Q969Z0 | -0.31 |
| **STXBP3** | syntaxin binding protein 3 | O00186 | -0.31 |
| **TOP1** | DNA topoisomerase I | P11387 | -0.309 |
| **RPL31** | ribosomal protein L31 | P62899 | -0.308 |
| **C1QBP** | complement C1q binding protein | Q07021 | -0.305 |
| **CAPZA1** | capping actin protein of muscle Z-line subunit alpha 1 | P52907 | -0.304 |
| **UBE2M** | ubiquitin conjugating enzyme E2 M | P61081 | -0.304 |
| **MARS** | methionyl-tRNA synthetase | P56192 | -0.303 |
| **HSP90AA1** | heat shock protein 90 alpha family class A member 1 | P07900 | -0.301 |
| **BRAT1** | BRCA1 associated ATM activator 1 | Q6PJG6 | -0.301 |
| **MOGS** | mannosyl-oligosaccharide glucosidase | Q13724 | -0.297 |
| **COMT** | catechol-O-methyltransferase | P21964 | -0.296 |
| **STT3A** | STT3 oligosaccharyltransferase complex catalytic subunit A | P46977 | -0.296 |
| **PRKCD** | protein kinase C delta | Q05655 | -0.295 |
| **UBL4A** | ubiquitin like 4A | P11441 | -0.292 |
| **RNF114** | ring finger protein 114 | Q9Y508 | -0.292 |
| **TMCO1** | transmembrane and coiled-coil domains 1 | Q9UM00 | -0.291 |
| **SEL1L** | SEL1L, ERAD E3 ligase adaptor subunit | Q9UBV2 | -0.29 |
| **NUDC** | nuclear distribution C, dynein complex regulator | Q9Y266 | -0.288 |
| **GRB2** | growth factor receptor bound protein 2 | P62993 | -0.288 |
| **SSBP1** | single stranded DNA binding protein 1 | Q04837 | -0.287 |
| **ERP44** | endoplasmic reticulum protein 44 | Q9BS26 | -0.287 |
| **PSMB4** | proteasome subunit beta 4 | P28070 | -0.285 |
| **CDC42** | cell division cycle 42 | P60953 | -0.285 |
| **SAR1A** | secretion associated Ras related GTPase 1A | Q9NR31 | -0.285 |
| **SEC24C** | SEC24 homolog C, COPII coat complex component | P53992 | -0.279 |
| **LIG1** | DNA ligase 1 | P18858 | -0.278 |
| **TRIM33** | tripartite motif containing 33 | Q9UPN9 | -0.278 |
| **CRIP2** | cysteine rich protein 2 | P52943 | -0.277 |
| **CC2D1A** | coiled-coil and C2 domain containing 1A | Q6P1N0 | -0.277 |
| **PITPNB** | phosphatidylinositol transfer protein beta | P48739 | -0.276 |
| **ZPR1** | ZPR1 zinc finger | O75312 | -0.276 |
| **ARFGAP2** | ADP ribosylation factor GTPase activating protein 2 | Q8N6H7 | -0.276 |
| **UPF1** | UPF1 RNA helicase and ATPase | Q92900 | -0.275 |
| **UNC13D** | unc-13 homolog D | Q70J99 | -0.274 |
| **IMPDH1** | inosine monophosphate dehydrogenase 1 | P20839 | -0.272 |
| **SFN** | stratifin | P31947 | -0.27 |
| **SPTBN1** | spectrin beta, non-erythrocytic 1 | Q01082 | -0.267 |
| **NTMT1** | N-terminal Xaa-Pro-Lys N-methyltransferase 1 | Q9BV86 | -0.266 |
| **GMPS** | guanine monophosphate synthase | P49915 | -0.265 |
| **ITIH2** | inter-alpha-trypsin inhibitor heavy chain 2 | P19823 | -0.265 |
| **SEC23IP** | SEC23 interacting protein | Q9Y6Y8 | -0.263 |
| **PAK2** | p21 (RAC1) activated kinase 2 | Q13177 | -0.262 |
| **NHLRC2** | NHL repeat containing 2 | Q8NBF2 | -0.262 |
| **SEC31A** | SEC31 homolog A, COPII coat complex component | O94979 | -0.26 |
| **GEMIN5** | gem nuclear organelle associated protein 5 | Q8TEQ6 | -0.26 |
| **GSTM3** | glutathione S-transferase mu 3 | P21266 | -0.259 |
| **ILF3** | interleukin enhancer binding factor 3 | Q12906 | -0.257 |
| **TPR** | translocated promoter region, nuclear basket protein | P12270 | -0.257 |
| **LRRC47** | leucine rich repeat containing 47 | Q8N1G4 | -0.256 |
| **EIF4A1** | eukaryotic translation initiation factor 4A1 | P60842 | -0.254 |
| **NRBP1** | nuclear receptor binding protein 1 | Q9UHY1 | -0.248 |
| **APEH** | acylaminoacyl-peptide hydrolase | P13798 | -0.247 |
| **RPL27A** | ribosomal protein L27a | P46776 | -0.246 |
| **RAD50** | RAD50 double strand break repair protein | Q92878 | -0.246 |
| **SEC62** | SEC62 homolog, preprotein translocation factor | Q99442 | -0.24 |
| **ARF5** | ADP ribosylation factor 5 | P84085 | -0.24 |
| **IMMT** | inner membrane mitochondrial protein | Q16891 | -0.239 |
| **GNB2** | G protein subunit beta 2 | P62879 | -0.238 |
| **RPL21** | ribosomal protein L21 | P46778 | -0.234 |
| **RPL18** | ribosomal protein L18 | Q07020 | -0.234 |
| **NT5DC1** | 5'-nucleotidase domain containing 1 | Q5TFE4 | -0.234 |
| **USP7** | ubiquitin specific peptidase 7 | Q93009 | -0.233 |
| **DDX6** | DEAD-box helicase 6 | P26196 | -0.231 |
| **AARS** | alanyl-tRNA synthetase | P49588 | -0.231 |
| **GPD2** | glycerol-3-phosphate dehydrogenase 2 | P43304 | -0.231 |
| **PDCD6IP** | programmed cell death 6 interacting protein | Q8WUM4 | -0.231 |
| **PRPSAP1** | phosphoribosyl pyrophosphate synthetase associated protein 1 | Q14558 | -0.23 |
| **ASNS** | asparagine synthetase (glutamine-hydrolyzing) | P08243 | -0.23 |
| **SELENBP1** | selenium binding protein 1 | Q13228 | -0.228 |
| **SLC25A13** | solute carrier family 25 member 13 | Q9UJS0 | -0.228 |
| **DPP3** | dipeptidyl peptidase 3 | Q9NY33 | -0.227 |
| **CAPRIN1** | cell cycle associated protein 1 | Q14444 | -0.227 |
| **MCCC1** | methylcrotonoyl-CoA carboxylase 1 | Q96RQ3 | -0.227 |
| **NDUFA13** | NADH:ubiquinone oxidoreductase subunit A13 | Q9P0J0 | -0.225 |
| **NPEPPS** | aminopeptidase puromycin sensitive | P55786 | -0.224 |
| **RAB35** | RAB35, member RAS oncogene family | Q15286 | -0.223 |
| **NAA50** | N(alpha)-acetyltransferase 50, NatE catalytic subunit | Q9GZZ1 | -0.223 |
| **TOMM70** | translocase of outer mitochondrial membrane 70 | O94826 | -0.223 |
| **RAC1** | Rac family small GTPase 1 | P63000 | -0.221 |
| **EPS8L2** | EPS8 like 2 | Q9H6S3 | -0.221 |
| **LLGL2** | LLGL scribble cell polarity complex component 2 | Q6P1M3 | -0.22 |
| **OXCT1** | 3-oxoacid CoA-transferase 1 | P55809 | -0.22 |
| **GTPBP4** | GTP binding protein 4 | Q9BZE4 | -0.217 |
| **ACAA2** | acetyl-CoA acyltransferase 2 | P42765 | -0.216 |
| **GRSF1** | G-rich RNA sequence binding factor 1 | Q12849 | -0.215 |
| **DCTPP1** | dCTP pyrophosphatase 1 | Q9H773 | -0.215 |
| **KRT80** | keratin 80 | Q6KB66 | -0.214 |
| **ATP6V1A** | ATPase H+ transporting V1 subunit A | P38606 | -0.214 |
| **CCT6A** | chaperonin containing TCP1 subunit 6A | P40227 | -0.212 |
| **ATP2B1** | ATPase plasma membrane Ca2+ transporting 1 | P20020 | -0.211 |
| **PSMB7** | proteasome subunit beta 7 | Q99436 | -0.211 |
| **EIF2A** | eukaryotic translation initiation factor 2A | Q9BY44 | -0.21 |
| **LARS** | leucyl-tRNA synthetase | Q9P2J5 | -0.209 |
| **ARPC4** | actin related protein 2/3 complex subunit 4 | P59998 | -0.207 |
| **SART3** | spliceosome associated factor 3, U4/U6 recycling protein | Q15020 | -0.205 |
| **TKT** | transketolase | P29401 | -0.204 |
| **TMEM165** | transmembrane protein 165 | Q9HC07 | -0.203 |
| **HCFC1** | host cell factor C1 | P51610 | -0.202 |
| **MYH9** | myosin heavy chain 9 | P35579 | -0.196 |
| **KRT7** | keratin 7 | P08729 | -0.196 |
| **COPB1** | coatomer protein complex subunit beta 1 | P53618 | -0.194 |
| **NCEH1** | neutral cholesterol ester hydrolase 1 | Q6PIU2 | -0.194 |
| **NAP1L4** | nucleosome assembly protein 1 like 4 | Q99733 | -0.193 |
| **RPL17** | ribosomal protein L17 | P18621 | -0.192 |
| **UFL1** | UFM1 specific ligase 1 | O94874 | -0.191 |
| **RPN1** | ribophorin I | P04843 | -0.188 |
| **EIF3A** | eukaryotic translation initiation factor 3 subunit A | Q14152 | -0.187 |
| **CLUH** | clustered mitochondria homolog | O75153 | -0.185 |
| **PC** | pyruvate carboxylase | P11498 | -0.182 |
| **RPL12** | ribosomal protein L12 | P30050 | -0.182 |
| **PSMD1** | proteasome 26S subunit, non-ATPase 1 | Q99460 | -0.181 |
| **FLII** | FLII actin remodeling protein | Q13045 | -0.181 |
| **NDUFS1** | NADH:ubiquinone oxidoreductase core subunit S1 | P28331 | -0.18 |
| **SMC4** | structural maintenance of chromosomes 4 | Q9NTJ3 | -0.179 |
| **HDLBP** | high density lipoprotein binding protein | Q00341 | -0.178 |
| **UBR5** | ubiquitin protein ligase E3 component n-recognin 5 | O95071 | -0.178 |
| **CTNND1** | catenin delta 1 | O60716 | -0.177 |
| **TRMT1** | tRNA methyltransferase 1 | Q9NXH9 | -0.177 |
| **PGAM1** | phosphoglycerate mutase 1 | P18669 | -0.176 |
| **MCM2** | minichromosome maintenance complex component 2 | P49736 | -0.175 |
| **PPID** | peptidylprolyl isomerase D | Q08752 | -0.175 |
| **MATR3** | matrin 3 | P43243 | -0.175 |
| **TLN1** | talin 1 | Q9Y490 | -0.172 |
| **PKM** | pyruvate kinase M1/2 | P14618 | -0.17 |
| **VCP** | valosin containing protein | P55072 | -0.17 |
| **EPS15L1** | epidermal growth factor receptor pathway substrate 15 like 1 | Q9UBC2 | -0.169 |
| **WASF2** | WAS protein family member 2 | Q9Y6W5 | -0.168 |
| **EPRS** | glutamyl-prolyl-tRNA synthetase | P07814 | -0.168 |
| **PSMD12** | proteasome 26S subunit, non-ATPase 12 | O00232 | -0.168 |
| **RPL3** | ribosomal protein L3 | P39023 | -0.168 |
| **ABCF3** | ATP binding cassette subfamily F member 3 | Q9NUQ8 | -0.167 |
| **NIT2** | nitrilase family member 2 | Q9NQR4 | -0.166 |
| **LAP3** | leucine aminopeptidase 3 | P28838 | -0.166 |
| **SSR4** | signal sequence receptor subunit 4 | P51571 | -0.165 |
| **EEF2** | eukaryotic translation elongation factor 2 | P13639 | -0.165 |
| **CARS** | cysteinyl-tRNA synthetase | P49589 | -0.164 |
| **RPL5** | ribosomal protein L5 | P46777 | -0.164 |
| **SEPT8** | septin 8 | Q92599 | -0.161 |
| **EIF3B** | eukaryotic translation initiation factor 3 subunit B | P55884 | -0.16 |
| **PDIA3** | protein disulfide isomerase family A member 3 | P30101 | -0.158 |
| **PFKP** | phosphofructokinase, platelet | Q01813 | -0.156 |
| **SLC7A1** | solute carrier family 7 member 1 | P30825 | -0.156 |
| **SMC1A** | structural maintenance of chromosomes 1A | Q14683 | -0.156 |
| **ATP5PB** | ATP synthase peripheral stalk-membrane subunit b | P24539 | -0.156 |
| **PHPT1** | phosphohistidine phosphatase 1 | Q9NRX4 | -0.156 |
| **PTGES3** | prostaglandin E synthase 3 | Q15185 | -0.155 |
| **COPE** | coatomer protein complex subunit epsilon | O14579 | -0.154 |
| **FAM129B** | family with sequence similarity 129 member B | Q96TA1 | -0.154 |
| **AGR2** | anterior gradient 2, protein disulphide isomerase family member | O95994 | -0.151 |
| **AP1B1** | adaptor related protein complex 1 subunit beta 1 | Q10567 | -0.148 |
| **HSP90B1** | heat shock protein 90 beta family member 1 | P14625 | -0.145 |
| **GCN1** | GCN1, eIF2 alpha kinase activator homolog | Q92616 | -0.145 |
| **NAPRT** | nicotinate phosphoribosyltransferase | Q6XQN6 | -0.143 |
| **EIF3L** | eukaryotic translation initiation factor 3 subunit L | Q9Y262 | -0.143 |
| **PSMD6** | proteasome 26S subunit, non-ATPase 6 | Q15008 | -0.143 |
| **GMPPB** | GDP-mannose pyrophosphorylase B | Q9Y5P6 | -0.142 |
| **PRPF6** | pre-mRNA processing factor 6 | O94906 | -0.141 |
| **EEF1G** | eukaryotic translation elongation factor 1 gamma | P26641 | -0.141 |
| **RNPEP** | arginyl aminopeptidase | Q9H4A4 | -0.14 |
| **NDUFS2** | NADH:ubiquinone oxidoreductase core subunit S2 | O75306 | -0.139 |
| **NPLOC4** | NPL4 homolog, ubiquitin recognition factor | Q8TAT6 | -0.139 |
| **TFRC** | transferrin receptor | P02786 | -0.138 |
| **DYNC1LI1** | dynein cytoplasmic 1 light intermediate chain 1 | Q9Y6G9 | -0.138 |
| **PLS3** | plastin 3 | P13797 | -0.136 |
| **FASN** | fatty acid synthase | P49327 | -0.136 |
| **ALDH1B1** | aldehyde dehydrogenase 1 family member B1 | P30837 | -0.134 |
| **LSM4** | LSM4 homolog, U6 small nuclear RNA and mRNA degradation associated | Q9Y4Z0 | -0.134 |
| **LCP1** | lymphocyte cytosolic protein 1 | P13796 | -0.133 |
| **ESYT1** | extended synaptotagmin 1 | Q9BSJ8 | -0.13 |
| **ATP5MG** | ATP synthase membrane subunit g | O75964 | -0.13 |
| **COASY** | Coenzyme A synthase | Q13057 | -0.129 |
| **STIP1** | stress induced phosphoprotein 1 | P31948 | -0.127 |
| **RPS7** | ribosomal protein S7 | P62081 | -0.125 |
| **PRKCSH** | protein kinase C substrate 80K-H | P14314 | -0.125 |
| **QARS** | glutaminyl-tRNA synthetase | P47897 | -0.124 |
| **RPS9** | ribosomal protein S9 | P46781 | -0.122 |
| **EIF2S3** | eukaryotic translation initiation factor 2 subunit gamma | P41091 | -0.121 |
| **LRPPRC** | leucine rich pentatricopeptide repeat containing | P42704 | -0.121 |
| **ATP1A1** | ATPase Na+/K+ transporting subunit alpha 1 | P05023 | -0.121 |
| **PRDX4** | peroxiredoxin 4 | Q13162 | -0.12 |
| **CCT7** | chaperonin containing TCP1 subunit 7 | Q99832 | -0.118 |
| **IDE** | insulin degrading enzyme | P14735 | -0.118 |
| **DYNC1H1** | dynein cytoplasmic 1 heavy chain 1 | Q14204 | -0.116 |
| **UGDH** | UDP-glucose 6-dehydrogenase | O60701 | -0.115 |
| **CFL1** | cofilin 1 | P23528 | -0.114 |
| **NMD3** | NMD3 ribosome export adaptor | Q96D46 | -0.113 |
| **ACADVL** | acyl-CoA dehydrogenase very long chain | P49748 | -0.112 |
| **COX4I1** | cytochrome c oxidase subunit 4I1 | P13073 | -0.111 |
| **ATG3** | autophagy related 3 | Q9NT62 | -0.11 |
| **SSB** | small RNA binding exonuclease protection factor La | P05455 | -0.109 |
| **SEPT2** | septin 2 | Q15019 | -0.108 |
| **TXNL1** | thioredoxin like 1 | O43396 | -0.108 |
| **HSPA5** | heat shock protein family A (Hsp70) member 5 | P11021 | -0.108 |
| **NAP1L1** | nucleosome assembly protein 1 like 1 | P55209 | -0.107 |
| **ATP6V1H** | ATPase H+ transporting V1 subunit H | Q9UI12 | -0.107 |
| **RPL11** | ribosomal protein L11 | P62913 | -0.106 |
| **RPL32** | ribosomal protein L32 | P62910 | -0.105 |
| **NUP88** | nucleoporin 88 | Q99567 | -0.105 |
| **DCAF7** | DDB1 and CUL4 associated factor 7 | P61962 | -0.104 |
| **AP1G1** | adaptor related protein complex 1 subunit gamma 1 | O43747 | -0.104 |
| **AAMP** | angio associated migratory cell protein | Q13685 | -0.101 |
| **UBE2L3** | ubiquitin conjugating enzyme E2 L3 | P68036 | -0.099 |
| **OGA** | O-GlcNAcase | O60502 | -0.098 |
| **TWF1** | twinfilin actin binding protein 1 | Q12792 | -0.091 |
| **FLNB** | filamin B | O75369 | -0.089 |
| **MTHFD1** | methylenetetrahydrofolate dehydrogenase, cyclohydrolase and formyltetrahydrofolate synthetase 1 | P11586 | -0.088 |
| **SCAMP3** | secretory carrier membrane protein 3 | O14828 | -0.086 |
| **OGFR** | opioid growth factor receptor | Q9NZT2 | -0.085 |
| **PPP6R3** | protein phosphatase 6 regulatory subunit 3 | Q5H9R7 | -0.085 |
| **PDLIM5** | PDZ and LIM domain 5 | Q96HC4 | -0.083 |
| **JUP** | junction plakoglobin | P14923 | -0.083 |
| **ARHGDIA** | Rho GDP dissociation inhibitor alpha | P52565 | -0.082 |
| **PTCD3** | pentatricopeptide repeat domain 3 | Q96EY7 | -0.079 |
| **EMC1** | ER membrane protein complex subunit 1 | Q8N766 | -0.079 |
| **CCT3** | chaperonin containing TCP1 subunit 3 | P49368 | -0.078 |
| **ARPC3** | actin related protein 2/3 complex subunit 3 | O15145 | -0.078 |
| **GLUD1** | glutamate dehydrogenase 1 | P00367 | -0.076 |
| **AFG3L2** | AFG3 like matrix AAA peptidase subunit 2 | Q9Y4W6 | -0.074 |
| **SUSD2** | sushi domain containing 2 | Q9UGT4 | -0.074 |
| **WARS** | tryptophanyl-tRNA synthetase | P23381 | -0.073 |
| **TXNDC5** | thioredoxin domain containing 5 | Q8NBS9 | -0.072 |
| **ESD** | esterase D | P10768 | -0.071 |
| **MYH10** | myosin heavy chain 10 | P35580 | -0.069 |
| **AIMP1** | aminoacyl tRNA synthetase complex interacting multifunctional protein 1 | Q12904 | -0.068 |
| **ANKFY1** | ankyrin repeat and FYVE domain containing 1 | Q9P2R3 | -0.065 |
| **ATP5MF** | ATP synthase membrane subunit f | P56134 | -0.064 |
| **RPS8** | ribosomal protein S8 | P62241 | -0.063 |
| **COPB2** | coatomer protein complex subunit beta 2 | P35606 | -0.063 |
| **SEC23B** | Sec23 homolog B, coat complex II component | Q15437 | -0.062 |
| **RRBP1** | ribosome binding protein 1 | Q9P2E9 | -0.062 |
| **LETM1** | leucine zipper and EF-hand containing transmembrane protein 1 | O95202 | -0.062 |
| **UBE2V1** | ubiquitin conjugating enzyme E2 V1 | Q13404 | -0.06 |
| **GPS1** | G protein pathway suppressor 1 | Q13098 | -0.06 |
| **HNRNPH2** | heterogeneous nuclear ribonucleoprotein H2 | P55795 | -0.059 |
| **PFKL** | phosphofructokinase, liver type | P17858 | -0.057 |
| **HARS** | histidyl-tRNA synthetase | P12081 | -0.055 |
| **RPL35** | ribosomal protein L35 | P42766 | -0.054 |
| **DNPEP** | aspartyl aminopeptidase | Q9ULA0 | -0.054 |
| **ATP5ME** | ATP synthase membrane subunit e | P56385 | -0.054 |
| **TACC2** | transforming acidic coiled-coil containing protein 2 | O95359 | -0.053 |
| **RPS17** | ribosomal protein S17 | P08708 | -0.052 |
| **AHNAK** | AHNAK nucleoprotein | Q09666 | -0.052 |
| **RPS14** | ribosomal protein S14 | P62263 | -0.051 |
| **RPL10A** | ribosomal protein L10a | P62906 | -0.051 |
| **NASP** | nuclear autoantigenic sperm protein | P49321 | -0.051 |
| **SRP19** | signal recognition particle 19 | P09132 | -0.051 |
| **EIF3E** | eukaryotic translation initiation factor 3 subunit E | P60228 | -0.05 |
| **RAP1GDS1** | Rap1 GTPase-GDP dissociation stimulator 1 | P52306 | -0.048 |
| **P4HA1** | prolyl 4-hydroxylase subunit alpha 1 | P13674 | -0.048 |
| **ADK** | adenosine kinase | P55263 | -0.047 |
| **GDI2** | GDP dissociation inhibitor 2 | P50395 | -0.046 |
| **PSMC4** | proteasome 26S subunit, ATPase 4 | P43686 | -0.046 |
| **RUVBL1** | RuvB like AAA ATPase 1 | Q9Y265 | -0.046 |
| **YWHAG** | tyrosine 3-monooxygenase/tryptophan 5-monooxygenase activation protein gamma | P61981 | -0.046 |
| **SNRPD3** | small nuclear ribonucleoprotein D3 polypeptide | P62318 | -0.044 |
| **VPS45** | vacuolar protein sorting 45 homolog | Q9NRW7 | -0.044 |
| **CNDP2** | carnosine dipeptidase 2 | Q96KP4 | -0.044 |
| **MLEC** | malectin | Q14165 | -0.043 |
| **NAA25** | N(alpha)-acetyltransferase 25, NatB auxiliary subunit | Q14CX7 | -0.04 |
| **PDCD4** | programmed cell death 4 | Q53EL6 | -0.04 |
| **TCOF1** | treacle ribosome biogenesis factor 1 | Q13428 | -0.039 |
| **ACTN1** | actinin alpha 1 | P12814 | -0.038 |
| **YARS** | tyrosyl-tRNA synthetase | P54577 | -0.037 |
| **AP2A1** | adaptor related protein complex 2 subunit alpha 1 | O95782 | -0.037 |
| **ACTN2** | actinin alpha 2 | P35609 | -0.036 |
| **LONP1** | lon peptidase 1, mitochondrial | P36776 | -0.035 |
| **HSPA4L** | heat shock protein family A (Hsp70) member 4 like | O95757 | -0.035 |
| **SCIN** | scinderin | Q9Y6U3 | -0.033 |
| **ACOT7** | acyl-CoA thioesterase 7 | O00154 | -0.033 |
| **APEX1** | apurinic/apyrimidinic endodeoxyribonuclease 1 | P27695 | -0.031 |
| **MAT2A** | methionine adenosyltransferase 2A | P31153 | -0.031 |
| **UGGT1** | UDP-glucose glycoprotein glucosyltransferase 1 | Q9NYU2 | -0.03 |
| **TRAPPC3** | trafficking protein particle complex 3 | O43617 | -0.03 |
| **GNS** | glucosamine (N-acetyl)-6-sulfatase | P15586 | -0.03 |
| **WBP11** | WW domain binding protein 11 | Q9Y2W2 | -0.028 |
| **ALDH7A1** | aldehyde dehydrogenase 7 family member A1 | P49419 | -0.027 |
| **ACO2** | aconitase 2 | Q99798 | -0.026 |
| **P4HB** | prolyl 4-hydroxylase subunit beta | P07237 | -0.026 |
| **LACTB2** | lactamase beta 2 | Q53H82 | -0.024 |
| **RAN** | RAN, member RAS oncogene family | P62826 | -0.024 |
| **GANAB** | glucosidase II alpha subunit | Q14697 | -0.022 |
| **PHB** | prohibitin | P35232 | -0.02 |
| **PSMB5** | proteasome subunit beta 5 | P28074 | -0.019 |
| **RPL37A** | ribosomal protein L37a | P61513 | -0.018 |
| **KYNU** | kynureninase | Q16719 | -0.018 |
| **PDS5A** | PDS5 cohesin associated factor A | Q29RF7 | -0.018 |
| **RPS15A** | ribosomal protein S15a | P62244 | -0.017 |
| **DDX21** | DExD-box helicase 21 | Q9NR30 | -0.012 |
| **RPS16** | ribosomal protein S16 | P62249 | -0.011 |
| **GALK1** | galactokinase 1 | P51570 | -0.011 |
| **RPN2** | ribophorin II | P04844 | -0.01 |
| **FAH** | fumarylacetoacetate hydrolase | P16930 | -0.008 |
| **MYO1C** | myosin IC | O00159 | -0.006 |
| **HADHA** | hydroxyacyl-CoA dehydrogenase trifunctional multienzyme complex subunit alpha | P40939 | -0.004 |
| **RPS5** | ribosomal protein S5 | P46782 | -0.003 |
| **RIDA** | reactive intermediate imine deaminase A homolog | P52758 | -0.002 |
| **PPA2** | pyrophosphatase (inorganic) 2 | Q9H2U2 | -0.001 |
| **APMAP** | adipocyte plasma membrane associated protein | Q9HDC9 | 0.001 |
| **CKAP5** | cytoskeleton associated protein 5 | Q14008 | 0.002 |
| **RPL14** | ribosomal protein L14 | P50914 | 0.004 |
| **EEF1A2** | eukaryotic translation elongation factor 1 alpha 2 | Q05639 | 0.005 |
| **STAT1** | signal transducer and activator of transcription 1 | P42224 | 0.005 |
| **MCM7** | minichromosome maintenance complex component 7 | P33993 | 0.007 |
| **GSPT1** | G1 to S phase transition 1 | P15170 | 0.008 |
| **NUP50** | nucleoporin 50 | Q9UKX7 | 0.01 |
| **ARCN1** | archain 1 | P48444 | 0.011 |
| **CTNNA1** | catenin alpha 1 | P35221 | 0.011 |
| **PSMC1** | proteasome 26S subunit, ATPase 1 | P62191 | 0.012 |
| **GALNT6** | polypeptide N-acetylgalactosaminyltransferase 6 | Q8NCL4 | 0.012 |
| **HSDL2** | hydroxysteroid dehydrogenase like 2 | Q6YN16 | 0.017 |
| **ATP6V1B2** | ATPase H+ transporting V1 subunit B2 | P21281 | 0.018 |
| **PIP4K2C** | phosphatidylinositol-5-phosphate 4-kinase type 2 gamma | Q8TBX8 | 0.019 |
| **HMGB1** | high mobility group box 1 | P09429 | 0.02 |
| **SRSF7** | serine and arginine rich splicing factor 7 | Q16629 | 0.02 |
| **OLA1** | Obg like ATPase 1 | Q9NTK5 | 0.021 |
| **FUBP1** | far upstream element binding protein 1 | Q96AE4 | 0.021 |
| **CTNNB1** | catenin beta 1 | P35222 | 0.021 |
| **CPNE1** | copine 1 | Q99829 | 0.021 |
| **CCT4** | chaperonin containing TCP1 subunit 4 | P50991 | 0.023 |
| **PBDC1** | polysaccharide biosynthesis domain containing 1 | Q9BVG4 | 0.023 |
| **FAF1** | Fas associated factor 1 | Q9UNN5 | 0.023 |
| **SETD3** | SET domain containing 3, actin histidine methyltransferase | Q86TU7 | 0.023 |
| **MCCC2** | methylcrotonoyl-CoA carboxylase 2 | Q9HCC0 | 0.024 |
| **PCMT1** | protein-L-isoaspartate (D-aspartate) O-methyltransferase | P22061 | 0.025 |
| **COPS3** | COP9 signalosome subunit 3 | Q9UNS2 | 0.025 |
| **SLC3A2** | solute carrier family 3 member 2 | P08195 | 0.025 |
| **DNAJA1** | DnaJ heat shock protein family (Hsp40) member A1 | P31689 | 0.027 |
| **SQSTM1** | sequestosome 1 | Q13501 | 0.028 |
| **ATP5PO** | ATP synthase peripheral stalk subunit OSCP | P48047 | 0.028 |
| **TRIM25** | tripartite motif containing 25 | Q14258 | 0.03 |
| **KPNB1** | karyopherin subunit beta 1 | Q14974 | 0.031 |
| **CAD** | carbamoyl-phosphate synthetase 2, aspartate transcarbamylase, and dihydroorotase | P27708 | 0.032 |
| **PTPN11** | protein tyrosine phosphatase, non-receptor type 11 | Q06124 | 0.032 |
| **HSPA9** | heat shock protein family A (Hsp70) member 9 | P38646 | 0.033 |
| **SCRN1** | secernin 1 | Q12765 | 0.033 |
| **MYO1B** | myosin IB | O43795 | 0.034 |
| **PRMT1** | protein arginine methyltransferase 1 | Q99873 | 0.036 |
| **RPL15** | ribosomal protein L15 | P61313 | 0.037 |
| **RARS** | arginyl-tRNA synthetase | P54136 | 0.039 |
| **G6PD** | glucose-6-phosphate dehydrogenase | P11413 | 0.039 |
| **AHSA1** | activator of HSP90 ATPase activity 1 | O95433 | 0.041 |
| **COPA** | coatomer protein complex subunit alpha | P53621 | 0.043 |
| **ACOX1** | acyl-CoA oxidase 1 | Q15067 | 0.045 |
| **SLC9A3R1** | SLC9A3 regulator 1 | O14745 | 0.045 |
| **ACAT1** | acetyl-CoA acetyltransferase 1 | P24752 | 0.045 |
| **LRRC59** | leucine rich repeat containing 59 | Q96AG4 | 0.045 |
| **HNRNPF** | heterogeneous nuclear ribonucleoprotein F | P52597 | 0.045 |
| **RAB10** | RAB10, member RAS oncogene family | P61026 | 0.049 |
| **FARSB** | phenylalanyl-tRNA synthetase subunit beta | Q9NSD9 | 0.049 |
| **CYCS** | cytochrome c, somatic | P99999 | 0.049 |
| **EIF4G1** | eukaryotic translation initiation factor 4 gamma 1 | Q04637 | 0.05 |
| **CTPS1** | CTP synthase 1 | P17812 | 0.052 |
| **G3BP2** | G3BP stress granule assembly factor 2 | Q9UN86 | 0.052 |
| **ATP2A3** | ATPase sarcoplasmic/endoplasmic reticulum Ca2+ transporting 3 | Q93084 | 0.053 |
| **KIF5B** | kinesin family member 5B | P33176 | 0.056 |
| **PMPCA** | peptidase, mitochondrial processing alpha subunit | Q10713 | 0.056 |
| **RBBP4** | RB binding protein 4, chromatin remodeling factor | Q09028 | 0.057 |
| **PSMD4** | proteasome 26S subunit, non-ATPase 4 | P55036 | 0.059 |
| **TBCD** | tubulin folding cofactor D | Q9BTW9 | 0.061 |
| **RPL6** | ribosomal protein L6 | Q02878 | 0.062 |
| **CNOT1** | CCR4-NOT transcription complex subunit 1 | A5YKK6 | 0.062 |
| **SHMT2** | serine hydroxymethyltransferase 2 | P34897 | 0.063 |
| **PA2G4** | proliferation-associated 2G4 | Q9UQ80 | 0.066 |
| **ACP1** | acid phosphatase 1 | P24666 | 0.066 |
| **PGD** | phosphogluconate dehydrogenase | P52209 | 0.066 |
| **ACTR2** | actin related protein 2 | P61160 | 0.069 |
| **NCAPG** | non-SMC condensin I complex subunit G | Q9BPX3 | 0.072 |
| **RAB7A** | RAB7A, member RAS oncogene family | P51149 | 0.072 |
| **EHD1** | EH domain containing 1 | Q9H4M9 | 0.072 |
| **UGP2** | UDP-glucose pyrophosphorylase 2 | Q16851 | 0.073 |
| **MRPL28** | mitochondrial ribosomal protein L28 | Q13084 | 0.073 |
| **SNRPE** | small nuclear ribonucleoprotein polypeptide E | P62304 | 0.073 |
| **NDUFV1** | NADH:ubiquinone oxidoreductase core subunit V1 | P49821 | 0.074 |
| **KIF1BP** | KIF1 binding protein | Q96EK5 | 0.074 |
| **PCNA** | proliferating cell nuclear antigen | P12004 | 0.074 |
| **ARFGEF3** | ARFGEF family member 3 | Q5TH69 | 0.075 |
| **SCRIB** | scribble planar cell polarity protein | Q14160 | 0.077 |
| **PPM1G** | protein phosphatase, Mg2+/Mn2+ dependent 1G | O15355 | 0.077 |
| **HYOU1** | hypoxia up-regulated 1 | Q9Y4L1 | 0.079 |
| **DDB1** | damage specific DNA binding protein 1 | Q16531 | 0.08 |
| **ADAR** | adenosine deaminase RNA specific | P55265 | 0.08 |
| **ACO1** | aconitase 1 | P21399 | 0.08 |
| **HSPA1A/HSPA1B** | heat shock protein family A (Hsp70) member 1A | P0DMV8 | 0.082 |
| **HMGB2** | high mobility group box 2 | P26583 | 0.082 |
| **NOP2** | NOP2 nucleolar protein | P46087 | 0.083 |
| **SRSF1** | serine and arginine rich splicing factor 1 | Q07955 | 0.084 |
| **PAFAH1B2** | platelet activating factor acetylhydrolase 1b catalytic subunit 2 | P68402 | 0.085 |
| **ENO1** | enolase 1 | P06733 | 0.085 |
| **GLA** | galactosidase alpha | P06280 | 0.086 |
| **RPS15** | ribosomal protein S15 | P62841 | 0.088 |
| **TUBB** | tubulin beta class I | P07437 | 0.089 |
| **GTF3C5** | general transcription factor IIIC subunit 5 | Q9Y5Q8 | 0.089 |
| **C12orf10** | chromosome 12 open reading frame 10 | Q9HB07 | 0.089 |
| **RPS25** | ribosomal protein S25 | P62851 | 0.09 |
| **PAFAH1B3** | platelet activating factor acetylhydrolase 1b catalytic subunit 3 | Q15102 | 0.091 |
| **UQCRC1** | ubiquinol-cytochrome c reductase core protein 1 | P31930 | 0.092 |
| **NLN** | neurolysin | Q9BYT8 | 0.092 |
| **RAB21** | RAB21, member RAS oncogene family | Q9UL25 | 0.092 |
| **IDH3A** | isocitrate dehydrogenase 3 (NAD(+)) alpha | P50213 | 0.093 |
| **NIPSNAP1** | nipsnap homolog 1 | Q9BPW8 | 0.093 |
| **LRRFIP1** | LRR binding FLII interacting protein 1 | Q32MZ4 | 0.094 |
| **BUB3** | BUB3 mitotic checkpoint protein | O43684 | 0.095 |
| **EIF3H** | eukaryotic translation initiation factor 3 subunit H | O15372 | 0.095 |
| **SARNP** | SAP domain containing ribonucleoprotein | P82979 | 0.098 |
| **SEC61B** | Sec61 translocon beta subunit | P60468 | 0.099 |
| **PGLS** | 6-phosphogluconolactonase | O95336 | 0.102 |
| **RPL4** | ribosomal protein L4 | P36578 | 0.102 |
| **RPL8** | ribosomal protein L8 | P62917 | 0.103 |
| **TSN** | translin | Q15631 | 0.104 |
| **RPS6KB1** | ribosomal protein S6 kinase B1 | P23443 | 0.104 |
| **RPL27** | ribosomal protein L27 | P61353 | 0.105 |
| **GOLGB1** | golgin B1 | Q14789 | 0.105 |
| **LYPLA1** | lysophospholipase 1 | O75608 | 0.105 |
| **PUF60** | poly(U) binding splicing factor 60 | Q9UHX1 | 0.106 |
| **TSTA3** | tissue specific transplantation antigen P35B | Q13630 | 0.108 |
| **TK1** | thymidine kinase 1 | P04183 | 0.111 |
| **NQO1** | NAD(P)H quinone dehydrogenase 1 | P15559 | 0.112 |
| **GFM1** | G elongation factor mitochondrial 1 | Q96RP9 | 0.115 |
| **NDRG1** | N-myc downstream regulated 1 | Q92597 | 0.116 |
| **TMX1** | thioredoxin related transmembrane protein 1 | Q9H3N1 | 0.118 |
| **STIM1** | stromal interaction molecule 1 | Q13586 | 0.12 |
| **HSD17B4** | hydroxysteroid 17-beta dehydrogenase 4 | P51659 | 0.12 |
| **PPIA** | peptidylprolyl isomerase A | P62937 | 0.12 |
| **VBP1** | VHL binding protein 1 | P61758 | 0.122 |
| **FOXK1** | forkhead box K1 | P85037 | 0.126 |
| **PABPC1** | poly(A) binding protein cytoplasmic 1 | P11940 | 0.128 |
| **CUL4A** | cullin 4A | Q13619 | 0.128 |
| **PSMA4** | proteasome subunit alpha 4 | P25789 | 0.128 |
| **IVL** | involucrin | P07476 | 0.129 |
| **PSMB1** | proteasome subunit beta 1 | P20618 | 0.132 |
| **ARHGAP1** | Rho GTPase activating protein 1 | Q07960 | 0.132 |
| **RPS24** | ribosomal protein S24 | P62847 | 0.132 |
| **RPL7A** | ribosomal protein L7a | P62424 | 0.134 |
| **RAB1A** | RAB1A, member RAS oncogene family | P62820 | 0.136 |
| **OPA1** | OPA1 mitochondrial dynamin like GTPase | O60313 | 0.136 |
| **HNRNPH3** | heterogeneous nuclear ribonucleoprotein H3 | P31942 | 0.137 |
| **CCT5** | chaperonin containing TCP1 subunit 5 | P48643 | 0.139 |
| **S100A9** | S100 calcium binding protein A9 | P06702 | 0.14 |
| **PARK7** | Parkinsonism associated deglycase | Q99497 | 0.14 |
| **DARS2** | aspartyl-tRNA synthetase 2, mitochondrial | Q6PI48 | 0.142 |
| **GAPVD1** | GTPase activating protein and VPS9 domains 1 | Q14C86 | 0.145 |
| **STK24** | serine/threonine kinase 24 | Q9Y6E0 | 0.145 |
| **NUCB1** | nucleobindin 1 | Q02818 | 0.147 |
| **PSMC3** | proteasome 26S subunit, ATPase 3 | P17980 | 0.147 |
| **DDOST** | dolichyl-diphosphooligosaccharide--protein glycosyltransferase non-catalytic subunit | P39656 | 0.149 |
| **TPT1** | tumor protein, translationally-controlled 1 | P13693 | 0.15 |
| **NUP155** | nucleoporin 155 | O75694 | 0.151 |
| **HSPA8** | heat shock protein family A (Hsp70) member 8 | P11142 | 0.156 |
| **AHCY** | adenosylhomocysteinase | P23526 | 0.156 |
| **HEXA** | hexosaminidase subunit alpha | P06865 | 0.158 |
| **SUGT1** | SGT1 homolog, MIS12 kinetochore complex assembly cochaperone | Q9Y2Z0 | 0.16 |
| **DBNL** | drebrin like | Q9UJU6 | 0.163 |
| **CAT** | catalase | P04040 | 0.165 |
| **ALDH16A1** | aldehyde dehydrogenase 16 family member A1 | Q8IZ83 | 0.165 |
| **PMPCB** | peptidase, mitochondrial processing beta subunit | O75439 | 0.165 |
| **GSTO1** | glutathione S-transferase omega 1 | P78417 | 0.168 |
| **NT5C2** | 5'-nucleotidase, cytosolic II | P49902 | 0.169 |
| **DLST** | dihydrolipoamide S-succinyltransferase | P36957 | 0.169 |
| **TTLL12** | tubulin tyrosine ligase like 12 | Q14166 | 0.17 |
| **ACAA1** | acetyl-CoA acyltransferase 1 | P09110 | 0.17 |
| **GNAI3** | G protein subunit alpha i3 | P08754 | 0.176 |
| **YWHAE** | tyrosine 3-monooxygenase/tryptophan 5-monooxygenase activation protein epsilon | P62258 | 0.177 |
| **VCL** | vinculin | P18206 | 0.177 |
| **CAPS** | calcyphosine | Q13938 | 0.178 |
| **ATP5F1A** | ATP synthase F1 subunit alpha | P25705 | 0.178 |
| **DDAH2** | dimethylarginine dimethylaminohydrolase 2 | O95865 | 0.179 |
| **RTN4** | reticulon 4 | Q9NQC3 | 0.179 |
| **PFDN1** | prefoldin subunit 1 | O60925 | 0.179 |
| **RNF213** | ring finger protein 213 | Q63HN8 | 0.18 |
| **HNRNPA0** | heterogeneous nuclear ribonucleoprotein A0 | Q13151 | 0.181 |
| **HSPA14** | heat shock protein family A (Hsp70) member 14 | Q0VDF9 | 0.182 |
| **AKR1A1** | aldo-keto reductase family 1 member A1 | P14550 | 0.182 |
| **LDHB** | lactate dehydrogenase B | P07195 | 0.183 |
| **ANXA5** | annexin A5 | P08758 | 0.183 |
| **APRT** | adenine phosphoribosyltransferase | P07741 | 0.184 |
| **LANCL1** | LanC like 1 | O43813 | 0.184 |
| **TPI1** | triosephosphate isomerase 1 | P60174 | 0.185 |
| **RPS6** | ribosomal protein S6 | P62753 | 0.185 |
| **GSN** | gelsolin | P06396 | 0.186 |
| **UQCRC2** | ubiquinol-cytochrome c reductase core protein 2 | P22695 | 0.186 |
| **CCT8** | chaperonin containing TCP1 subunit 8 | P50990 | 0.191 |
| **COPG1** | coatomer protein complex subunit gamma 1 | Q9Y678 | 0.191 |
| **AP2B1** | adaptor related protein complex 2 subunit beta 1 | P63010 | 0.192 |
| **NDUFS3** | NADH:ubiquinone oxidoreductase core subunit S3 | O75489 | 0.192 |
| **DAD1** | defender against cell death 1 | P61803 | 0.195 |
| **RACK1** | receptor for activated C kinase 1 | P63244 | 0.195 |
| **PRPF31** | pre-mRNA processing factor 31 | Q8WWY3 | 0.197 |
| **HSPBP1** | HSPA (Hsp70) binding protein 1 | Q9NZL4 | 0.198 |
| **SNX3** | sorting nexin 3 | O60493 | 0.198 |
| **BANF1** | barrier to autointegration factor 1 | O75531 | 0.198 |
| **RAB18** | RAB18, member RAS oncogene family | Q9NP72 | 0.198 |
| **PSMD8** | proteasome 26S subunit, non-ATPase 8 | P48556 | 0.2 |
| **FEN1** | flap structure-specific endonuclease 1 | P39748 | 0.201 |
| **PUS7** | pseudouridine synthase 7 | Q96PZ0 | 0.203 |
| **PCBD1** | pterin-4 alpha-carbinolamine dehydratase 1 | P61457 | 0.204 |
| **PSMA7** | proteasome subunit alpha 7 | O14818 | 0.204 |
| **KRT17** | keratin 17 | Q04695 | 0.204 |
| **IVD** | isovaleryl-CoA dehydrogenase | P26440 | 0.207 |
| **AK2** | adenylate kinase 2 | P54819 | 0.208 |
| **BZW2** | basic leucine zipper and W2 domains 2 | Q9Y6E2 | 0.209 |
| **MSH2** | mutS homolog 2 | P43246 | 0.211 |
| **MSH6** | mutS homolog 6 | P52701 | 0.212 |
| **HNRNPA1** | heterogeneous nuclear ribonucleoprotein A1 | P09651 | 0.212 |
| **DERL1** | derlin 1 | Q9BUN8 | 0.214 |
| **ELOB** | elongin B | Q15370 | 0.214 |
| **GSR** | glutathione-disulfide reductase | P00390 | 0.216 |
| **RAB2A** | RAB2A, member RAS oncogene family | P61019 | 0.216 |
| **TMED9** | transmembrane p24 trafficking protein 9 | Q9BVK6 | 0.218 |
| **ATP5F1C** | ATP synthase F1 subunit gamma | P36542 | 0.222 |
| **PLOD2** | procollagen-lysine,2-oxoglutarate 5-dioxygenase 2 | O00469 | 0.222 |
| **PLIN3** | perilipin 3 | O60664 | 0.223 |
| **RPS3** | ribosomal protein S3 | P23396 | 0.223 |
| **RANBP1** | RAN binding protein 1 | P43487 | 0.225 |
| **SNRNP200** | small nuclear ribonucleoprotein U5 subunit 200 | O75643 | 0.225 |
| **LDHA** | lactate dehydrogenase A | P00338 | 0.225 |
| **SERPINH1** | serpin family H member 1 | P50454 | 0.226 |
| **SIN3A** | SIN3 transcription regulator family member A | Q96ST3 | 0.227 |
| **MACROD1** | mono-ADP ribosylhydrolase 1 | Q9BQ69 | 0.228 |
| **ALDH9A1** | aldehyde dehydrogenase 9 family member A1 | P49189 | 0.23 |
| **VPS35** | VPS35 retromer complex component | Q96QK1 | 0.23 |
| **HUWE1** | HECT, UBA and WWE domain containing 1, E3 ubiquitin protein ligase | Q7Z6Z7 | 0.231 |
| **ISOC1** | isochorismatase domain containing 1 | Q96CN7 | 0.231 |
| **CORO1B** | coronin 1B | Q9BR76 | 0.232 |
| **MRPL23** | mitochondrial ribosomal protein L23 | Q16540 | 0.232 |
| **PTBP1** | polypyrimidine tract binding protein 1 | P26599 | 0.233 |
| **CCT2** | chaperonin containing TCP1 subunit 2 | P78371 | 0.235 |
| **RPS3A** | ribosomal protein S3A | P61247 | 0.236 |
| **GGH** | gamma-glutamyl hydrolase | Q92820 | 0.236 |
| **ARL6IP5** | ADP ribosylation factor like GTPase 6 interacting protein 5 | O75915 | 0.237 |
| **RPS10** | ribosomal protein S10 | P46783 | 0.238 |
| **CCDC47** | coiled-coil domain containing 47 | Q96A33 | 0.239 |
| **TBCB** | tubulin folding cofactor B | Q99426 | 0.239 |
| **CLTC** | clathrin heavy chain | Q00610 | 0.24 |
| **PSME1** | proteasome activator subunit 1 | Q06323 | 0.243 |
| **NAMPT** | nicotinamide phosphoribosyltransferase | P43490 | 0.247 |
| **PSMD11** | proteasome 26S subunit, non-ATPase 11 | O00231 | 0.249 |
| **ACTR3** | actin related protein 3 | P61158 | 0.249 |
| **TIMM44** | translocase of inner mitochondrial membrane 44 | O43615 | 0.251 |
| **MVP** | major vault protein | Q14764 | 0.251 |
| **PSMB3** | proteasome subunit beta 3 | P49720 | 0.251 |
| **PSMA2** | proteasome subunit alpha 2 | P25787 | 0.252 |
| **DNAJC7** | DnaJ heat shock protein family (Hsp40) member C7 | Q99615 | 0.252 |
| **MTA2** | metastasis associated 1 family member 2 | O94776 | 0.253 |
| **RAP2C** | RAP2C, member of RAS oncogene family | Q9Y3L5 | 0.256 |
| **GRPEL1** | GrpE like 1, mitochondrial | Q9HAV7 | 0.256 |
| **TUFM** | Tu translation elongation factor, mitochondrial | P49411 | 0.256 |
| **BCAP31** | B cell receptor associated protein 31 | P51572 | 0.26 |
| **PSMA1** | proteasome subunit alpha 1 | P25786 | 0.26 |
| **ALDH6A1** | aldehyde dehydrogenase 6 family member A1 | Q02252 | 0.263 |
| **ANXA11** | annexin A11 | P50995 | 0.263 |
| **GPD1L** | glycerol-3-phosphate dehydrogenase 1 like | Q8N335 | 0.265 |
| **SCAMP2** | secretory carrier membrane protein 2 | O15127 | 0.265 |
| **RUVBL2** | RuvB like AAA ATPase 2 | Q9Y230 | 0.266 |
| **TMED10** | transmembrane p24 trafficking protein 10 | P49755 | 0.269 |
| **UNC45A** | unc-45 myosin chaperone A | Q9H3U1 | 0.269 |
| **HADH** | hydroxyacyl-CoA dehydrogenase | Q16836 | 0.27 |
| **PDIA6** | protein disulfide isomerase family A member 6 | Q15084 | 0.27 |
| **POR** | cytochrome p450 oxidoreductase | P16435 | 0.271 |
| **DDI2** | DNA damage inducible 1 homolog 2 | Q5TDH0 | 0.271 |
| **IRGQ** | immunity related GTPase Q | Q8WZA9 | 0.272 |
| **PSMA3** | proteasome subunit alpha 3 | P25788 | 0.273 |
| **PPP1CA** | protein phosphatase 1 catalytic subunit alpha | P62136 | 0.279 |
| **PRKAR1A** | protein kinase cAMP-dependent type I regulatory subunit alpha | P10644 | 0.28 |
| **CSTB** | cystatin B | P04080 | 0.281 |
| **GPI** | glucose-6-phosphate isomerase | P06744 | 0.281 |
| **PPA1** | pyrophosphatase (inorganic) 1 | Q15181 | 0.281 |
| **SPR** | sepiapterin reductase | P35270 | 0.281 |
| **ERBB2** | erb-b2 receptor tyrosine kinase 2 | P04626 | 0.282 |
| **TUBB4A** | tubulin beta 4A class IVa | P04350 | 0.284 |
| **CLPTM1** | CLPTM1 regulator of GABA type A receptor forward trafficking | O96005 | 0.285 |
| **PPIF** | peptidylprolyl isomerase F | P30405 | 0.286 |
| **FLNA** | filamin A | P21333 | 0.287 |
| **PRDX6** | peroxiredoxin 6 | P30041 | 0.287 |
| **PSME2** | proteasome activator subunit 2 | Q9UL46 | 0.29 |
| **GORASP2** | golgi reassembly stacking protein 2 | Q9H8Y8 | 0.29 |
| **PFAS** | phosphoribosylformylglycinamidine synthase | O15067 | 0.291 |
| **ECHS1** | enoyl-CoA hydratase, short chain 1 | P30084 | 0.291 |
| **DRG2** | developmentally regulated GTP binding protein 2 | P55039 | 0.291 |
| **PRMT5** | protein arginine methyltransferase 5 | O14744 | 0.292 |
| **ASL** | argininosuccinate lyase | P04424 | 0.293 |
| **UFD1** | ubiquitin recognition factor in ER associated degradation 1 | Q92890 | 0.294 |
| **RPL23A** | ribosomal protein L23a | P62750 | 0.295 |
| **XPO7** | exportin 7 | Q9UIA9 | 0.296 |
| **SUCLA2** | succinate-CoA ligase ADP-forming beta subunit | Q9P2R7 | 0.296 |
| **CAPZA2** | capping actin protein of muscle Z-line subunit alpha 2 | P47755 | 0.298 |
| **PDXK** | pyridoxal kinase | O00764 | 0.298 |
| **ETFA** | electron transfer flavoprotein subunit alpha | P13804 | 0.3 |
| **PDXDC1** | pyridoxal dependent decarboxylase domain containing 1 | Q6P996 | 0.301 |
| **SLC25A5** | solute carrier family 25 member 5 | P05141 | 0.302 |
| **KRT4** | keratin 4 | P19013 | 0.303 |
| **INTS3** | integrator complex subunit 3 | Q68E01 | 0.303 |
| **VAPB** | VAMP associated protein B and C | O95292 | 0.303 |
| **CS** | citrate synthase | O75390 | 0.304 |
| **THEM6** | thioesterase superfamily member 6 | Q8WUY1 | 0.305 |
| **ILF2** | interleukin enhancer binding factor 2 | Q12905 | 0.305 |
| **GFPT1** | glutamine--fructose-6-phosphate transaminase 1 | Q06210 | 0.306 |
| **BCCIP** | BRCA2 and CDKN1A interacting protein | Q9P287 | 0.306 |
| **KHSRP** | KH-type splicing regulatory protein | Q92945 | 0.306 |
| **IMPDH2** | inosine monophosphate dehydrogenase 2 | P12268 | 0.308 |
| **ATP5F1B** | ATP synthase F1 subunit beta | P06576 | 0.313 |
| **PSMA5** | proteasome subunit alpha 5 | P28066 | 0.317 |
| **PSMB2** | proteasome subunit beta 2 | P49721 | 0.318 |
| **STAU1** | staufen double-stranded RNA binding protein 1 | O95793 | 0.318 |
| **TST** | thiosulfate sulfurtransferase | Q16762 | 0.319 |
| **VDAC2** | voltage dependent anion channel 2 | P45880 | 0.319 |
| **GBF1** | golgi brefeldin A resistant guanine nucleotide exchange factor 1 | Q92538 | 0.32 |
| **PPME1** | protein phosphatase methylesterase 1 | Q9Y570 | 0.32 |
| **SLC25A3** | solute carrier family 25 member 3 | Q00325 | 0.321 |
| **EIF4G3** | eukaryotic translation initiation factor 4 gamma 3 | O43432 | 0.322 |
| **COX6C** | cytochrome c oxidase subunit 6C | P09669 | 0.323 |
| **OTUB1** | OTU deubiquitinase, ubiquitin aldehyde binding 1 | Q96FW1 | 0.323 |
| **TUBB4B** | tubulin beta 4B class IVb | P68371 | 0.327 |
| **GNB1** | G protein subunit beta 1 | P62873 | 0.328 |
| **SRPK1** | SRSF protein kinase 1 | Q96SB4 | 0.329 |
| **CAPZB** | capping actin protein of muscle Z-line subunit beta | P47756 | 0.331 |
| **GGCT** | gamma-glutamylcyclotransferase | O75223 | 0.332 |
| **PSME3** | proteasome activator subunit 3 | P61289 | 0.333 |
| **YWHAQ** | tyrosine 3-monooxygenase/tryptophan 5-monooxygenase activation protein theta | P27348 | 0.334 |
| **PDHA1** | pyruvate dehydrogenase E1 alpha 1 subunit | P08559 | 0.336 |
| **EIF3F** | eukaryotic translation initiation factor 3 subunit F | O00303 | 0.336 |
| **PHB2** | prohibitin 2 | Q99623 | 0.336 |
| **KPNA3** | karyopherin subunit alpha 3 | O00505 | 0.338 |
| **HM13** | histocompatibility minor 13 | Q8TCT9 | 0.338 |
| **HPDL** | 4-hydroxyphenylpyruvate dioxygenase like | Q96IR7 | 0.342 |
| **LGALS3BP** | galectin 3 binding protein | Q08380 | 0.342 |
| **SUMF2** | sulfatase modifying factor 2 | Q8NBJ7 | 0.344 |
| **VDAC1** | voltage dependent anion channel 1 | P21796 | 0.346 |
| **VASP** | vasodilator stimulated phosphoprotein | P50552 | 0.351 |
| **RAB1B** | RAB1B, member RAS oncogene family | Q9H0U4 | 0.352 |
| **PRKACA** | protein kinase cAMP-activated catalytic subunit alpha | P17612 | 0.352 |
| **PRDX2** | peroxiredoxin 2 | P32119 | 0.353 |
| **KARS** | lysyl-tRNA synthetase | Q15046 | 0.353 |
| **VAT1** | vesicle amine transport 1 | Q99536 | 0.356 |
| **GLOD4** | glyoxalase domain containing 4 | Q9HC38 | 0.356 |
| **TUBB6** | tubulin beta 6 class V | Q9BUF5 | 0.356 |
| **FUBP3** | far upstream element binding protein 3 | Q96I24 | 0.356 |
| **CUL3** | cullin 3 | Q13618 | 0.357 |
| **TUBA1C** | tubulin alpha 1c | Q9BQE3 | 0.357 |
| **ABRAXAS2** | abraxas 2, BRISC complex subunit | Q15018 | 0.358 |
| **TATDN1** | TatD DNase domain containing 1 | Q6P1N9 | 0.358 |
| **STOML2** | stomatin like 2 | Q9UJZ1 | 0.36 |
| **CMPK1** | cytidine/uridine monophosphate kinase 1 | P30085 | 0.36 |
| **DLD** | dihydrolipoamide dehydrogenase | P09622 | 0.362 |
| **RPS4X** | ribosomal protein S4 X-linked | P62701 | 0.363 |
| **IRF3** | interferon regulatory factor 3 | Q14653 | 0.363 |
| **CYC1** | cytochrome c1 | P08574 | 0.364 |
| **DECR1** | 2,4-dienoyl-CoA reductase 1 | Q16698 | 0.366 |
| **HIBADH** | 3-hydroxyisobutyrate dehydrogenase | P31937 | 0.367 |
| **EPPK1** | epiplakin 1 | P58107 | 0.367 |
| **CIAPIN1** | cytokine induced apoptosis inhibitor 1 | Q6FI81 | 0.367 |
| **TOMM22** | translocase of outer mitochondrial membrane 22 | Q9NS69 | 0.368 |
| **KRT18** | keratin 18 | P05783 | 0.37 |
| **KRT8** | keratin 8 | P05787 | 0.371 |
| **STRAP** | serine/threonine kinase receptor associated protein | Q9Y3F4 | 0.371 |
| **NUDT5** | nudix hydrolase 5 | Q9UKK9 | 0.371 |
| **AIMP2** | aminoacyl tRNA synthetase complex interacting multifunctional protein 2 | Q13155 | 0.374 |
| **RAB14** | RAB14, member RAS oncogene family | P61106 | 0.374 |
| **PSMD14** | proteasome 26S subunit, non-ATPase 14 | O00487 | 0.375 |
| **DLAT** | dihydrolipoamide S-acetyltransferase | P10515 | 0.375 |
| **HSPD1** | heat shock protein family D (Hsp60) member 1 | P10809 | 0.376 |
| **NDUFA2** | NADH:ubiquinone oxidoreductase subunit A2 | O43678 | 0.376 |
| **RPL18A** | ribosomal protein L18a | Q02543 | 0.377 |
| **RPS2** | ribosomal protein S2 | P15880 | 0.383 |
| **CBR1** | carbonyl reductase 1 | P16152 | 0.385 |
| **TRIP13** | thyroid hormone receptor interactor 13 | Q15645 | 0.387 |
| **CAP1** | cyclase associated actin cytoskeleton regulatory protein 1 | Q01518 | 0.39 |
| **CDC73** | cell division cycle 73 | Q6P1J9 | 0.391 |
| **UFC1** | ubiquitin-fold modifier conjugating enzyme 1 | Q9Y3C8 | 0.391 |
| **TMEM43** | transmembrane protein 43 | Q9BTV4 | 0.392 |
| **YWHAZ** | tyrosine 3-monooxygenase/tryptophan 5-monooxygenase activation protein zeta | P63104 | 0.393 |
| **FAHD1** | fumarylacetoacetate hydrolase domain containing 1 | Q6P587 | 0.395 |
| **TAGLN2** | transgelin 2 | P37802 | 0.396 |
| **KDM1A** | lysine demethylase 1A | O60341 | 0.4 |
| **FBP1** | fructose-bisphosphatase 1 | P09467 | 0.401 |
| **STUB1** | STIP1 homology and U-box containing protein 1 | Q9UNE7 | 0.407 |
| **CACYBP** | calcyclin binding protein | Q9HB71 | 0.407 |
| **SOD2** | superoxide dismutase 2 | P04179 | 0.408 |
| **HADHB** | hydroxyacyl-CoA dehydrogenase trifunctional multienzyme complex subunit beta | P55084 | 0.409 |
| **ABCF1** | ATP binding cassette subfamily F member 1 | Q8NE71 | 0.412 |
| **ABCF2** | ATP binding cassette subfamily F member 2 | Q9UG63 | 0.413 |
| **HNRNPM** | heterogeneous nuclear ribonucleoprotein M | P52272 | 0.413 |
| **NAPA** | NSF attachment protein alpha | P54920 | 0.413 |
| **MDH1** | malate dehydrogenase 1 | P40925 | 0.415 |
| **ABAT** | 4-aminobutyrate aminotransferase | P80404 | 0.416 |
| **ACLY** | ATP citrate lyase | P53396 | 0.418 |
| **MAPRE1** | microtubule associated protein RP/EB family member 1 | Q15691 | 0.419 |
| **PURB** | purine rich element binding protein B | Q96QR8 | 0.419 |
| **SLC25A10** | solute carrier family 25 member 10 | Q9UBX3 | 0.42 |
| **LSS** | lanosterol synthase | P48449 | 0.421 |
| **RPLP0** | ribosomal protein lateral stalk subunit P0 | P05388 | 0.422 |
| **SRPRB** | SRP receptor subunit beta | Q9Y5M8 | 0.422 |
| **TPBG** | trophoblast glycoprotein | Q13641 | 0.422 |
| **EEF1D** | eukaryotic translation elongation factor 1 delta | P29692 | 0.424 |
| **PGK1** | phosphoglycerate kinase 1 | P00558 | 0.424 |
| **RPL7** | ribosomal protein L7 | P18124 | 0.425 |
| **MTDH** | metadherin | Q86UE4 | 0.425 |
| **HSD17B12** | hydroxysteroid 17-beta dehydrogenase 12 | Q53GQ0 | 0.425 |
| **DDRGK1** | DDRGK domain containing 1 | Q96HY6 | 0.428 |
| **RBM14** | RNA binding motif protein 14 | Q96PK6 | 0.429 |
| **SH3BGRL** | SH3 domain binding glutamate rich protein like | O75368 | 0.43 |
| **HIP1R** | huntingtin interacting protein 1 related | O75146 | 0.431 |
| **SEC23A** | Sec23 homolog A, coat complex II component | Q15436 | 0.431 |
| **ABHD12** | abhydrolase domain containing 12 | Q8N2K0 | 0.432 |
| **TOMM40** | translocase of outer mitochondrial membrane 40 | O96008 | 0.432 |
| **ANP32E** | acidic nuclear phosphoprotein 32 family member E | Q9BTT0 | 0.435 |
| **ANXA7** | annexin A7 | P20073 | 0.436 |
| **SAE1** | SUMO1 activating enzyme subunit 1 | Q9UBE0 | 0.437 |
| **MDH2** | malate dehydrogenase 2 | P40926 | 0.438 |
| **SERPINB1** | serpin family B member 1 | P30740 | 0.438 |
| **FTSJ3** | FtsJ RNA 2'-O-methyltransferase 3 | Q8IY81 | 0.439 |
| **PPIB** | peptidylprolyl isomerase B | P23284 | 0.441 |
| **ALDOA** | aldolase, fructose-bisphosphate A | P04075 | 0.441 |
| **PCBP1** | poly(rC) binding protein 1 | Q15365 | 0.444 |
| **COTL1** | coactosin like F-actin binding protein 1 | Q14019 | 0.445 |
| **ACTR1A** | actin related protein 1A | P61163 | 0.445 |
| **FARSA** | phenylalanyl-tRNA synthetase subunit alpha | Q9Y285 | 0.446 |
| **PSMA6** | proteasome subunit alpha 6 | P60900 | 0.449 |
| **PRDX1** | peroxiredoxin 1 | Q06830 | 0.45 |
| **ECI1** | enoyl-CoA delta isomerase 1 | P42126 | 0.451 |
| **RRP12** | ribosomal RNA processing 12 homolog | Q5JTH9 | 0.451 |
| **CUTA** | cutA divalent cation tolerance homolog | O60888 | 0.454 |
| **HMBS** | hydroxymethylbilane synthase | P08397 | 0.454 |
| **PHGDH** | phosphoglycerate dehydrogenase | O43175 | 0.456 |
| **HDGF** | heparin binding growth factor | P51858 | 0.458 |
| **LAMTOR1** | late endosomal/lysosomal adaptor, MAPK and MTOR activator 1 | Q6IAA8 | 0.459 |
| **CTBP2** | C-terminal binding protein 2 | P56545 | 0.46 |
| **ILK** | integrin linked kinase | Q13418 | 0.464 |
| **ERP29** | endoplasmic reticulum protein 29 | P30040 | 0.465 |
| **UBAP2L** | ubiquitin associated protein 2 like | Q14157 | 0.466 |
| **OSTF1** | osteoclast stimulating factor 1 | Q92882 | 0.469 |
| **RAB5C** | RAB5C, member RAS oncogene family | P51148 | 0.469 |
| **QSOX2** | quiescin sulfhydryl oxidase 2 | Q6ZRP7 | 0.47 |
| **GOT2** | glutamic-oxaloacetic transaminase 2 | P00505 | 0.471 |
| **MRPL12** | mitochondrial ribosomal protein L12 | P52815 | 0.475 |
| **SLC9A3R2** | SLC9A3 regulator 2 | Q15599 | 0.475 |
| **SRSF11** | serine and arginine rich splicing factor 11 | Q05519 | 0.477 |
| **SUB1** | SUB1 homolog, transcriptional regulator | P53999 | 0.477 |
| **NSDHL** | NAD(P) dependent steroid dehydrogenase-like | Q15738 | 0.478 |
| **ATXN2L** | ataxin 2 like | Q8WWM7 | 0.478 |
| **IDH2** | isocitrate dehydrogenase (NADP(+)) 2, mitochondrial | P48735 | 0.479 |
| **CNPY2** | canopy FGF signaling regulator 2 | Q9Y2B0 | 0.479 |
| **EIF4B** | eukaryotic translation initiation factor 4B | P23588 | 0.48 |
| **TPD52L1** | TPD52 like 1 | Q16890 | 0.48 |
| **TARDBP** | TAR DNA binding protein | Q13148 | 0.481 |
| **RPSA** | ribosomal protein SA | P08865 | 0.483 |
| **KRT19** | keratin 19 | P08727 | 0.483 |
| **SLC25A24** | solute carrier family 25 member 24 | Q6NUK1 | 0.484 |
| **PRPSAP2** | phosphoribosyl pyrophosphate synthetase associated protein 2 | O60256 | 0.488 |
| **PRDX3** | peroxiredoxin 3 | P30048 | 0.489 |
| **U2AF1/U2AF1L5** | U2 small nuclear RNA auxiliary factor 1 | Q01081 | 0.49 |
| **STAT3** | signal transducer and activator of transcription 3 | P40763 | 0.49 |
| **COX6B1** | cytochrome c oxidase subunit 6B1 | P14854 | 0.49 |
| **EXOC4** | exocyst complex component 4 | Q96A65 | 0.491 |
| **INF2** | inverted formin, FH2 and WH2 domain containing | Q27J81 | 0.493 |
| **SHTN1** | shootin 1 | A0MZ66 | 0.495 |
| **UMPS** | uridine monophosphate synthetase | P11172 | 0.495 |
| **ACAD9** | acyl-CoA dehydrogenase family member 9 | Q9H845 | 0.496 |
| **RPL13** | ribosomal protein L13 | P26373 | 0.496 |
| **PSMD7** | proteasome 26S subunit, non-ATPase 7 | P51665 | 0.498 |
| **USP15** | ubiquitin specific peptidase 15 | Q9Y4E8 | 0.501 |
| **ECH1** | enoyl-CoA hydratase 1 | Q13011 | 0.501 |
| **RHOA** | ras homolog family member A | P61586 | 0.504 |
| **TUBB3** | tubulin beta 3 class III | Q13509 | 0.507 |
| **PTPA** | protein phosphatase 2 phosphatase activator | Q15257 | 0.507 |
| **TALDO1** | transaldolase 1 | P37837 | 0.509 |
| **WDR1** | WD repeat domain 1 | O75083 | 0.511 |
| **PTRH2** | peptidyl-tRNA hydrolase 2 | Q9Y3E5 | 0.512 |
| **SEC16A** | SEC16 homolog A, endoplasmic reticulum export factor | O15027 | 0.513 |
| **MRPL15** | mitochondrial ribosomal protein L15 | Q9P015 | 0.513 |
| **RPL35A** | ribosomal protein L35a | P18077 | 0.516 |
| **PFN1** | profilin 1 | P07737 | 0.524 |
| **RAB5A** | RAB5A, member RAS oncogene family | P20339 | 0.527 |
| **GAPDH** | glyceraldehyde-3-phosphate dehydrogenase | P04406 | 0.527 |
| **CCDC124** | coiled-coil domain containing 124 | Q96CT7 | 0.528 |
| **MRPS27** | mitochondrial ribosomal protein S27 | Q92552 | 0.531 |
| **EIF3I** | eukaryotic translation initiation factor 3 subunit I | Q13347 | 0.531 |
| **DFFA** | DNA fragmentation factor subunit alpha | O00273 | 0.533 |
| **DDX42** | DEAD-box helicase 42 | Q86XP3 | 0.536 |
| **CPS1** | carbamoyl-phosphate synthase 1 | P31327 | 0.536 |
| **NPM1** | nucleophosmin 1 | P06748 | 0.537 |
| **CNOT2** | CCR4-NOT transcription complex subunit 2 | Q9NZN8 | 0.538 |
| **SUCLG2** | succinate-CoA ligase GDP-forming beta subunit | Q96I99 | 0.538 |
| **PRPF8** | pre-mRNA processing factor 8 | Q6P2Q9 | 0.543 |
| **TPM4** | tropomyosin 4 | P67936 | 0.543 |
| **ADSS** | adenylosuccinate synthase | P30520 | 0.544 |
| **TKFC** | triokinase and FMN cyclase | Q3LXA3 | 0.549 |
| **TPD52L2** | TPD52 like 2 | O43399 | 0.551 |
| **PSMB6** | proteasome subunit beta 6 | P28072 | 0.558 |
| **TBCA** | tubulin folding cofactor A | O75347 | 0.56 |
| **EEF1B2** | eukaryotic translation elongation factor 1 beta 2 | P24534 | 0.562 |
| **ELAVL1** | ELAV like RNA binding protein 1 | Q15717 | 0.565 |
| **LARP4** | La ribonucleoprotein domain family member 4 | Q71RC2 | 0.566 |
| **DNAJB1** | DnaJ heat shock protein family (Hsp40) member B1 | P25685 | 0.567 |
| **CTSD** | cathepsin D | P07339 | 0.568 |
| **REEP5** | receptor accessory protein 5 | Q00765 | 0.569 |
| **ATP6V1E1** | ATPase H+ transporting V1 subunit E1 | P36543 | 0.569 |
| **EIF2S1** | eukaryotic translation initiation factor 2 subunit alpha | P05198 | 0.571 |
| **SLC25A1** | solute carrier family 25 member 1 | P53007 | 0.577 |
| **STARD10** | StAR related lipid transfer domain containing 10 | Q9Y365 | 0.578 |
| **CSK** | C-terminal Src kinase | P41240 | 0.584 |
| **NANS** | N-acetylneuraminate synthase | Q9NR45 | 0.59 |
| **ETFB** | electron transfer flavoprotein subunit beta | P38117 | 0.591 |
| **SERPINB6** | serpin family B member 6 | P35237 | 0.592 |
| **NIF3L1** | NGG1 interacting factor 3 like 1 | Q9GZT8 | 0.593 |
| **PRDX5** | peroxiredoxin 5 | P30044 | 0.594 |
| **PSMD9** | proteasome 26S subunit, non-ATPase 9 | O00233 | 0.596 |
| **YWHAH** | tyrosine 3-monooxygenase/tryptophan 5-monooxygenase activation protein eta | Q04917 | 0.597 |
| **CDC5L** | cell division cycle 5 like | Q99459 | 0.598 |
| **PRPF19** | pre-mRNA processing factor 19 | Q9UMS4 | 0.598 |
| **YWHAB** | tyrosine 3-monooxygenase/tryptophan 5-monooxygenase activation protein beta | P31946 | 0.604 |
| **PSMC2** | proteasome 26S subunit, ATPase 2 | P35998 | 0.613 |
| **SMS** | spermine synthase | P52788 | 0.614 |
| **SRP14** | signal recognition particle 14 | P37108 | 0.614 |
| **ELOC** | elongin C | Q15369 | 0.616 |
| **TOR1AIP1** | torsin 1A interacting protein 1 | Q5JTV8 | 0.617 |
| **SNX5** | sorting nexin 5 | Q9Y5X3 | 0.619 |
| **DPYSL2** | dihydropyrimidinase like 2 | Q16555 | 0.62 |
| **NDUFB10** | NADH:ubiquinone oxidoreductase subunit B10 | O96000 | 0.621 |
| **ABHD11** | abhydrolase domain containing 11 | Q8NFV4 | 0.624 |
| **SRSF6** | serine and arginine rich splicing factor 6 | Q13247 | 0.626 |
| **ATP1B3** | ATPase Na+/K+ transporting subunit beta 3 | P54709 | 0.626 |
| **PRRC1** | proline rich coiled-coil 1 | Q96M27 | 0.63 |
| **ATL3** | atlastin GTPase 3 | Q6DD88 | 0.649 |
| **BZW1** | basic leucine zipper and W2 domains 1 | Q7L1Q6 | 0.65 |
| **IPO9** | importin 9 | Q96P70 | 0.656 |
| **SNAP29** | synaptosome associated protein 29 | O95721 | 0.657 |
| **MTCH2** | mitochondrial carrier 2 | Q9Y6C9 | 0.659 |
| **MRPS5** | mitochondrial ribosomal protein S5 | P82675 | 0.659 |
| **PNP** | purine nucleoside phosphorylase | P00491 | 0.672 |
| **LASP1** | LIM and SH3 protein 1 | Q14847 | 0.674 |
| **PGP** | phosphoglycolate phosphatase | A6NDG6 | 0.675 |
| **BTF3** | basic transcription factor 3 | P20290 | 0.675 |
| **PCK2** | phosphoenolpyruvate carboxykinase 2, mitochondrial | Q16822 | 0.678 |
| **UCHL3** | ubiquitin C-terminal hydrolase L3 | P15374 | 0.681 |
| **COPS4** | COP9 signalosome subunit 4 | Q9BT78 | 0.683 |
| **ADSL** | adenylosuccinate lyase | P30566 | 0.689 |
| **ZMPSTE24** | zinc metallopeptidase STE24 | O75844 | 0.689 |
| **COX5B** | cytochrome c oxidase subunit 5B | P10606 | 0.691 |
| **NAXE** | NAD(P)HX epimerase | Q8NCW5 | 0.695 |
| **TES** | testin LIM domain protein | Q9UGI8 | 0.697 |
| **IDH3B** | isocitrate dehydrogenase 3 (NAD(+)) beta | O43837 | 0.701 |
| **HMGB3** | high mobility group box 3 | O15347 | 0.702 |
| **GPHN** | gephyrin | Q9NQX3 | 0.703 |
| **GOT1** | glutamic-oxaloacetic transaminase 1 | P17174 | 0.703 |
| **CERS2** | ceramide synthase 2 | Q96G23 | 0.703 |
| **S100A11** | S100 calcium binding protein A11 | P31949 | 0.704 |
| **DCTN2** | dynactin subunit 2 | Q13561 | 0.705 |
| **FH** | fumarate hydratase | P07954 | 0.707 |
| **ALDH4A1** | aldehyde dehydrogenase 4 family member A1 | P30038 | 0.707 |
| **OAT** | ornithine aminotransferase | P04181 | 0.716 |
| **PSMC5** | proteasome 26S subunit, ATPase 5 | P62195 | 0.717 |
| **MRPS28** | mitochondrial ribosomal protein S28 | Q9Y2Q9 | 0.719 |
| **LAD1** | ladinin 1 | O00515 | 0.724 |
| **TPP2** | tripeptidyl peptidase 2 | P29144 | 0.725 |
| **CTSZ** | cathepsin Z | Q9UBR2 | 0.727 |
| **AP2M1** | adaptor related protein complex 2 subunit mu 1 | Q96CW1 | 0.73 |
| **BLVRA** | biliverdin reductase A | P53004 | 0.731 |
| **ANP32A** | acidic nuclear phosphoprotein 32 family member A | P39687 | 0.733 |
| **CDV3** | CDV3 homolog | Q9UKY7 | 0.733 |
| **HSPB1** | heat shock protein family B (small) member 1 | P04792 | 0.736 |
| **PTBP2** | polypyrimidine tract binding protein 2 | Q9UKA9 | 0.737 |
| **PFN2** | profilin 2 | P35080 | 0.753 |
| **WIPF2** | WAS/WASL interacting protein family member 2 | Q8TF74 | 0.757 |
| **PYM1** | PYM homolog 1, exon junction complex associated factor | Q9BRP8 | 0.76 |
| **PCBP2** | poly(rC) binding protein 2 | Q15366 | 0.76 |
| **HSD17B10** | hydroxysteroid 17-beta dehydrogenase 10 | Q99714 | 0.76 |
| **TOMM34** | translocase of outer mitochondrial membrane 34 | Q15785 | 0.764 |
| **SEPHS1** | selenophosphate synthetase 1 | P49903 | 0.765 |
| **NUDT9** | nudix hydrolase 9 | Q9BW91 | 0.766 |
| **PICALM** | phosphatidylinositol binding clathrin assembly protein | Q13492 | 0.772 |
| **H1F0** | H1 histone family member 0 | P07305 | 0.776 |
| **PFDN2** | prefoldin subunit 2 | Q9UHV9 | 0.777 |
| **CRKL** | CRK like proto-oncogene, adaptor protein | P46109 | 0.78 |
| **LAMP1** | lysosomal associated membrane protein 1 | P11279 | 0.785 |
| **PPP3CA** | protein phosphatase 3 catalytic subunit alpha | Q08209 | 0.786 |
| **BLVRB** | biliverdin reductase B | P30043 | 0.788 |
| **NDUFA9** | NADH:ubiquinone oxidoreductase subunit A9 | Q16795 | 0.789 |
| **LXN** | latexin | Q9BS40 | 0.79 |
| **RPS28** | ribosomal protein S28 | P62857 | 0.79 |
| **SMARCC2** | SWI/SNF related, matrix associated, actin dependent regulator of chromatin subfamily c member 2 | Q8TAQ2 | 0.792 |
| **USO1** | USO1 vesicle transport factor | O60763 | 0.797 |
| **ALDH18A1** | aldehyde dehydrogenase 18 family member A1 | P54886 | 0.8 |
| **DUT** | deoxyuridine triphosphatase | P33316 | 0.8 |
| **TPM3** | tropomyosin 3 | P06753 | 0.802 |
| **ANXA4** | annexin A4 | P09525 | 0.806 |
| **ATP5PD** | ATP synthase peripheral stalk subunit d | O75947 | 0.815 |
| **ATXN10** | ataxin 10 | Q9UBB4 | 0.818 |
| **RPLP1** | ribosomal protein lateral stalk subunit P1 | P05386 | 0.822 |
| **HINT1** | histidine triad nucleotide binding protein 1 | P49773 | 0.823 |
| **HIST1H1B** | histone cluster 1 H1 family member b | P16401 | 0.827 |
| **COPS2** | COP9 signalosome subunit 2 | P61201 | 0.831 |
| **ACAT2** | acetyl-CoA acetyltransferase 2 | Q9BWD1 | 0.835 |
| **GLO1** | glyoxalase I | Q04760 | 0.838 |
| **DDT** | D-dopachrome tautomerase | P30046 | 0.838 |
| **SNX6** | sorting nexin 6 | Q9UNH7 | 0.843 |
| **FAM120A** | family with sequence similarity 120A | Q9NZB2 | 0.846 |
| **SH3GL1** | SH3 domain containing GRB2 like 1, endophilin A2 | Q99961 | 0.848 |
| **HPRT1** | hypoxanthine phosphoribosyltransferase 1 | P00492 | 0.849 |
| **ANP32B** | acidic nuclear phosphoprotein 32 family member B | Q92688 | 0.849 |
| **CRYZ** | crystallin zeta | Q08257 | 0.855 |
| **NFS1** | NFS1 cysteine desulfurase | Q9Y697 | 0.863 |
| **SEC22B** | SEC22 homolog B, vesicle trafficking protein (gene/pseudogene) | O75396 | 0.864 |
| **SPATS2L** | spermatogenesis associated serine rich 2 like | Q9NUQ6 | 0.869 |
| **PGM3** | phosphoglucomutase 3 | O95394 | 0.883 |
| **IDI1** | isopentenyl-diphosphate delta isomerase 1 | Q13907 | 0.885 |
| **PSMC6** | proteasome 26S subunit, ATPase 6 | P62333 | 0.886 |
| **PDHB** | pyruvate dehydrogenase E1 beta subunit | P11177 | 0.894 |
| **LMAN2** | lectin, mannose binding 2 | Q12907 | 0.901 |
| **SUPT5H** | SPT5 homolog, DSIF elongation factor subunit | O00267 | 0.904 |
| **CTTN** | cortactin | Q14247 | 0.909 |
| **HNRNPAB** | heterogeneous nuclear ribonucleoprotein A/B | Q99729 | 0.91 |
| **ACADM** | acyl-CoA dehydrogenase medium chain | P11310 | 0.91 |
| **DEK** | DEK proto-oncogene | P35659 | 0.913 |
| **TPM1** | tropomyosin 1 | P09493 | 0.914 |
| **PNPO** | pyridoxamine 5'-phosphate oxidase | Q9NVS9 | 0.914 |
| **EIF2B1** | eukaryotic translation initiation factor 2B subunit alpha | Q14232 | 0.918 |
| **DNAJC9** | DnaJ heat shock protein family (Hsp40) member C9 | Q8WXX5 | 0.924 |
| **SRI** | sorcin | P30626 | 0.927 |
| **DCXR** | dicarbonyl and L-xylulose reductase | Q7Z4W1 | 0.933 |
| **GBA** | glucosylceramidase beta | P04062 | 0.934 |
| **IDH1** | isocitrate dehydrogenase (NADP(+)) 1, cytosolic | O75874 | 0.934 |
| **THOC3** | THO complex 3 | Q96J01 | 0.934 |
| **MCM5** | minichromosome maintenance complex component 5 | P33992 | 0.956 |
| **INPP4B** | inositol polyphosphate-4-phosphatase type II B | O15327 | 0.958 |
| **ASS1** | argininosuccinate synthase 1 | P00966 | 0.963 |
| **PPP1R12A** | protein phosphatase 1 regulatory subunit 12A | O14974 | 0.963 |
| **PDCD5** | programmed cell death 5 | O14737 | 0.967 |
| **NME1** | NME/NM23 nucleoside diphosphate kinase 1 | P15531 | 0.968 |
| **NAGK** | N-acetylglucosamine kinase | Q9UJ70 | 0.979 |
| **SFXN1** | sideroflexin 1 | Q9H9B4 | 0.98 |
| **HMOX2** | heme oxygenase 2 | P30519 | 0.983 |
| **RAD23B** | RAD23 homolog B, nucleotide excision repair protein | P54727 | 0.992 |
| **FABP5** | fatty acid binding protein 5 | Q01469 | 1 |
| **LIMA1** | LIM domain and actin binding 1 | Q9UHB6 | 1.003 |
| **CLNS1A** | chloride nucleotide-sensitive channel 1A | P54105 | 1.003 |
| **STX4** | syntaxin 4 | Q12846 | 1.004 |
| **ALDOC** | aldolase, fructose-bisphosphate C | P09972 | 1.004 |
| **GOLT1B** | golgi transport 1B | Q9Y3E0 | 1.012 |
| **EIF2B4** | eukaryotic translation initiation factor 2B subunit delta | Q9UI10 | 1.022 |
| **MIF** | macrophage migration inhibitory factor | P14174 | 1.028 |
| **NSF** | N-ethylmaleimide sensitive factor, vesicle fusing ATPase | P46459 | 1.039 |
| **PSMD13** | proteasome 26S subunit, non-ATPase 13 | Q9UNM6 | 1.054 |
| **BID** | BH3 interacting domain death agonist | P55957 | 1.056 |
| **S100P** | S100 calcium binding protein P | P25815 | 1.06 |
| **HNRNPC** | heterogeneous nuclear ribonucleoprotein C (C1/C2) | P07910 | 1.067 |
| **LARP1** | La ribonucleoprotein domain family member 1 | Q6PKG0 | 1.075 |
| **ZYX** | zyxin | Q15942 | 1.079 |
| **NUDCD1** | NudC domain containing 1 | Q96RS6 | 1.086 |
| **TMOD3** | tropomodulin 3 | Q9NYL9 | 1.089 |
| **UBQLN2** | ubiquilin 2 | Q9UHD9 | 1.089 |
| **PPP5C** | protein phosphatase 5 catalytic subunit | P53041 | 1.103 |
| **JPT1** | Jupiter microtubule associated homolog 1 | Q9UK76 | 1.106 |
| **TXN** | thioredoxin | P10599 | 1.138 |
| **NNT** | nicotinamide nucleotide transhydrogenase | Q13423 | 1.14 |
| **RRM2** | ribonucleotide reductase regulatory subunit M2 | P31350 | 1.141 |
| **HDHD5** | haloacid dehalogenase like hydrolase domain containing 5 | Q9BXW7 | 1.145 |
| **BPNT1** | 3'(2'), 5'-bisphosphate nucleotidase 1 | O95861 | 1.154 |
| **MYL6** | myosin light chain 6 | P60660 | 1.154 |
| **DNPH1** | 2'-deoxynucleoside 5'-phosphate N-hydrolase 1 | O43598 | 1.155 |
| **PPT1** | palmitoyl-protein thioesterase 1 | P50897 | 1.157 |
| **ANXA3** | annexin A3 | P12429 | 1.165 |
| **PGM1** | phosphoglucomutase 1 | P36871 | 1.17 |
| **CAPG** | capping actin protein, gelsolin like | P40121 | 1.185 |
| **RPL29** | ribosomal protein L29 | P47914 | 1.186 |
| **TXNDC17** | thioredoxin domain containing 17 | Q9BRA2 | 1.201 |
| **FKBP3** | FKBP prolyl isomerase 3 | Q00688 | 1.204 |
| **RFC4** | replication factor C subunit 4 | P35249 | 1.228 |
| **RPS21** | ribosomal protein S21 | P63220 | 1.233 |
| **PFDN6** | prefoldin subunit 6 | O15212 | 1.253 |
| **SPTLC1** | serine palmitoyltransferase long chain base subunit 1 | O15269 | 1.263 |
| **COX5A** | cytochrome c oxidase subunit 5A | P20674 | 1.265 |
| **PCYT2** | phosphate cytidylyltransferase 2, ethanolamine | Q99447 | 1.27 |
| **SLC25A6** | solute carrier family 25 member 6 | P12236 | 1.277 |
| **TPD52** | tumor protein D52 | P55327 | 1.278 |
| **HAT1** | histone acetyltransferase 1 | O14929 | 1.285 |
| **CSRP1** | cysteine and glycine rich protein 1 | P21291 | 1.295 |
| **PCYT1A** | phosphate cytidylyltransferase 1, choline, alpha | P49585 | 1.304 |
| **NDUFA4** | NDUFA4 mitochondrial complex associated | O00483 | 1.321 |
| **SNX27** | sorting nexin family member 27 | Q96L92 | 1.321 |
| **AK3** | adenylate kinase 3 | Q9UIJ7 | 1.336 |
| **GIPC1** | GIPC PDZ domain containing family member 1 | O14908 | 1.339 |
| **DHCR7** | 7-dehydrocholesterol reductase | Q9UBM7 | 1.347 |
| **STMN1** | stathmin 1 | P16949 | 1.403 |
| **TFG** | TRK-fused gene | Q92734 | 1.463 |
| **RPL34** | ribosomal protein L34 | P49207 | 1.469 |
| **RAE1** | ribonucleic acid export 1 | P78406 | 1.474 |
| **SRP54** | signal recognition particle 54 | P61011 | 1.477 |
| **FAU** | FAU ubiquitin like and ribosomal protein S30 fusion | P62861 | 1.483 |
| **CBR3** | carbonyl reductase 3 | O75828 | 1.484 |
| **SLC7A5** | solute carrier family 7 member 5 | Q01650 | 1.493 |
| **EEF1E1** | eukaryotic translation elongation factor 1 epsilon 1 | O43324 | 1.493 |
| **EIF3G** | eukaryotic translation initiation factor 3 subunit G | O75821 | 1.514 |
| **ADRM1** | adhesion regulating molecule 1 | Q16186 | 1.518 |
| **EPB41L1** | erythrocyte membrane protein band 4.1 like 1 | Q9H4G0 | 1.52 |
| **UFM1** | ubiquitin fold modifier 1 | P61960 | 1.526 |
| **SGTA** | small glutamine rich tetratricopeptide repeat containing alpha | O43765 | 1.528 |
| **PDAP1** | PDGFA associated protein 1 | Q13442 | 1.537 |
| **ADNP** | activity dependent neuroprotector homeobox | Q9H2P0 | 1.602 |
| **MTREX** | Mtr4 exosome RNA helicase | P42285 | 1.609 |
| **FDXR** | ferredoxin reductase | P22570 | 1.633 |
| **RBM25** | RNA binding motif protein 25 | P49756 | 1.635 |
| **OXSR1** | oxidative stress responsive kinase 1 | O95747 | 1.657 |
| **AKR1C3** | aldo-keto reductase family 1 member C3 | P42330 | 1.667 |
| **HSPE1** | heat shock protein family E (Hsp10) member 1 | P61604 | 1.675 |
| **RALA** | RAS like proto-oncogene A | P11233 | 1.691 |
| **MISP** | mitotic spindle positioning | Q8IVT2 | 1.701 |
| **PTPN1** | protein tyrosine phosphatase, non-receptor type 1 | P18031 | 1.701 |
| **NUTF2** | nuclear transport factor 2 | P61970 | 1.703 |
| **CDH1** | cadherin 1 | P12830 | 1.704 |
| **PDLIM1** | PDZ and LIM domain 1 | O00151 | 1.709 |
| **PGAM5** | PGAM family member 5, mitochondrial serine/threonine protein phosphatase | Q96HS1 | 1.712 |
| **MVD** | mevalonate diphosphate decarboxylase | P53602 | 1.742 |
| **BASP1** | brain abundant membrane attached signal protein 1 | P80723 | 1.747 |
| **UBXN1** | UBX domain protein 1 | Q04323 | 1.765 |
| **PPCS** | phosphopantothenoylcysteine synthetase | Q9HAB8 | 1.77 |
| **ETF1** | eukaryotic translation termination factor 1 | P62495 | 1.776 |
| **MAPK3** | mitogen-activated protein kinase 3 | P27361 | 1.787 |
| **VAPA** | VAMP associated protein A | Q9P0L0 | 1.792 |
| **HMGCS1** | 3-hydroxy-3-methylglutaryl-CoA synthase 1 | Q01581 | 1.832 |
| **TIMM50** | translocase of inner mitochondrial membrane 50 | Q3ZCQ8 | 1.864 |
| **ASNA1** | arsA arsenite transporter, ATP-binding, homolog 1 (bacterial) | O43681 | 1.872 |
| **EIF4A2** | eukaryotic translation initiation factor 4A2 | Q14240 | 1.906 |
| **BAX** | BCL2 associated X, apoptosis regulator | Q07812 | 2.015 |
| **NFKB2** | nuclear factor kappa B subunit 2 | Q00653 | 2.018 |
| **CHMP4B** | charged multivesicular body protein 4B | Q9H444 | 2.022 |
| **FAM3C** | family with sequence similarity 3 member C | Q92520 | 2.063 |
| **PNN** | pinin, desmosome associated protein | Q9H307 | 2.079 |
| **CCAR1** | cell division cycle and apoptosis regulator 1 | Q8IX12 | 2.199 |
| **RPLP2** | ribosomal protein lateral stalk subunit P2 | P05387 | 2.248 |
| **CHCHD3** | coiled-coil-helix-coiled-coil-helix domain containing 3 | Q9NX63 | 2.285 |
| **BCAT2** | branched chain amino acid transaminase 2 | O15382 | 2.351 |
| **MARCKS** | myristoylated alanine rich protein kinase C substrate | P29966 | 2.397 |
| **GOLGA2** | golgin A2 | Q08379 | 2.504 |
| **DBI** | diazepam binding inhibitor, acyl-CoA binding protein | P07108 | 2.537 |
| **ARHGEF16** | Rho guanine nucleotide exchange factor 16 | Q5VV41 | 2.704 |
| **RHEB** | Ras homolog, mTORC1 binding | Q15382 | 2.709 |
| **OTUD6B** | OTU domain containing 6B | Q8N6M0 | 2.827 |
| **UBXN4** | UBX domain protein 4 | Q92575 | 2.836 |
| **ZC3HAV1** | zinc finger CCCH-type containing, antiviral 1 | Q7Z2W4 | 2.943 |
| **ILVBL** | ilvB acetolactate synthase like | A1L0T0 | 3.036 |
| **SNW1** | SNW domain containing 1 | Q13573 | 3.159 |
| **HDHD3** | haloacid dehalogenase like hydrolase domain containing 3 | Q9BSH5 | 3.273 |
| **CAPN2** | calpain 2 | P17655 | 3.895 |
| **NEBL** | nebulette | O76041 | 5 |
| **JPT2** | Jupiter microtubule associated homolog 2 | Q9H910 | 5 |
| **PRRC2C** | proline rich coiled-coil 2C | Q9Y520 | 5 |
| **OVCA2** | OVCA2 serine hydrolase domain containing | Q8WZ82 | 5 |
| **SRRM2** | serine/arginine repetitive matrix 2 | Q9UQ35 | 5 |
| **GRB7** | growth factor receptor bound protein 7 | Q14451 | 5 |
| **TRMT1L** | tRNA methyltransferase 1 like | Q7Z2T5 | 5 |
| **PEPD** | peptidase D | P12955 | 5 |
| **PURA** | purine rich element binding protein A | Q00577 | 5 |
| **KRT16** | keratin 16 | P08779 | 5 |
| **NUCKS1** | nuclear casein kinase and cyclin dependent kinase substrate 1 | Q9H1E3 | 5 |
| **S100A16** | S100 calcium binding protein A16 | Q96FQ6 | 5 |
| **FKBP1A** | FKBP prolyl isomerase 1A | P62942 | 5 |
| **CSTF2** | cleavage stimulation factor subunit 2 | P33240 | 5 |
| **APOL2** | apolipoprotein L2 | Q9BQE5 | 5 |
| **AP3D1** | adaptor related protein complex 3 subunit delta 1 | O14617 | 5 |
| **TRPS1** | transcriptional repressor GATA binding 1 | Q9UHF7 | 5 |
| **TMPO** | thymopoietin | P42166 | 5 |
| **TMEM65** | transmembrane protein 65 | Q6PI78 | 5 |
| **MARCKSL1** | MARCKS like 1 | P49006 | 5 |
| **FDPS** | farnesyl diphosphate synthase | P14324 | 5 |
| **SCP2** | sterol carrier protein 2 | P22307 | 5 |
| **PDS5B** | PDS5 cohesin associated factor B | Q9NTI5 | 5 |
| **ADH5** | alcohol dehydrogenase 5 (class III), chi polypeptide | P11766 | 5 |
| **DNAJC2** | DnaJ heat shock protein family (Hsp40) member C2 | Q99543 | 5 |
| **UAP1L1** | UDP-N-acetylglucosamine pyrophosphorylase 1 like 1 | Q3KQV9 | 5 |
| **STX7** | syntaxin 7 | O15400 | 5 |
| **PRPF3** | pre-mRNA processing factor 3 | O43395 | 5 |
| **RANBP3** | RAN binding protein 3 | Q9H6Z4 | 5 |
| **AKR7A2** | aldo-keto reductase family 7 member A2 | O43488 | 5 |
| **PIR** | pirin | O00625 | 5 |
| **HTRA2** | HtrA serine peptidase 2 | O43464 | 5 |
| **MTHFD2** | methylenetetrahydrofolate dehydrogenase (NADP+ dependent) 2, methenyltetrahydrofolate cyclohydrolase | P13995 | 5 |
| **NPEPL1** | aminopeptidase like 1 | Q8NDH3 | 5 |
| **CYP1B1** | cytochrome P450 family 1 subfamily B member 1 | Q16678 | 5 |
| **PLPBP** | pyridoxal phosphate binding protein | O94903 | 5 |
| **PAXX** | PAXX non-homologous end joining factor | Q9BUH6 | 5 |
| **ESYT2** | extended synaptotagmin 2 | A0FGR8 | 5 |
| **H1FX** | H1 histone family member X | Q92522 | 6.623 |
| **TWF2** | twinfilin actin binding protein 2 | Q6IBS0 |  |
| **AP3B1** | adaptor related protein complex 3 subunit beta 1 | O00203 |  |
| **ABCD1** | ATP binding cassette subfamily D member 1 | P33897 |  |
| **TEX264** | testis expressed 264 | Q9Y6I9 |  |
| **B3GAT3** | beta-1,3-glucuronyltransferase 3 | O94766 |  |
| **GALE** | UDP-galactose-4-epimerase | Q14376 |  |
| **EVPL** | envoplakin | Q92817 |  |
| **S100A6** | S100 calcium binding protein A6 | P06703 |  |
| **ANXA1** | annexin A1 | P04083 |  |
